# T-helper cell phenotypes are repeatable, positively correlated, and associated with helminth infection in wild Soay sheep

**DOI:** 10.1093/discim/kyae017

**Published:** 2025-02-08

**Authors:** Yolanda Corripio-Miyar, Adam D Hayward, Hannah Lemon, Xavier Bal, Cameron Cunnea, Fiona Kenyon, Jill G Pilkington, Josephine M Pemberton, Daniel H Nussey, Tom N McNeilly

**Affiliations:** Moredun Research Institute, Pentlands Science Park, Penicuik, Midlothian, UK; Moredun Research Institute, Pentlands Science Park, Penicuik, Midlothian, UK; Institute of Ecology and Evolution, School of Biological Sciences, University of Edinburgh, Ashworth Laboratories, Edinburgh, UK; Department of Biology, University of Oxford, Oxford, UK; Institute of Ecology and Evolution, School of Biological Sciences, University of Edinburgh, Ashworth Laboratories, Edinburgh, UK; Moredun Research Institute, Pentlands Science Park, Penicuik, Midlothian, UK; Moredun Research Institute, Pentlands Science Park, Penicuik, Midlothian, UK; Institute of Ecology and Evolution, School of Biological Sciences, University of Edinburgh, Ashworth Laboratories, Edinburgh, UK; Institute of Ecology and Evolution, School of Biological Sciences, University of Edinburgh, Ashworth Laboratories, Edinburgh, UK; Institute of Ecology and Evolution, School of Biological Sciences, University of Edinburgh, Ashworth Laboratories, Edinburgh, UK; Moredun Research Institute, Pentlands Science Park, Penicuik, Midlothian, UK

**Keywords:** Cytokines, cell mediated immunity, nematodes and/or helminths, Th1, Th2

## Abstract

**Background:**

T-helper (Th) cells co-ordinate immune responses to ensure that infections with diverse parasites are controlled effectively. Helminth parasites such as gastrointestinal nematodes (GIN) are generally associated with T-helper type 2 (Th2) responses, while intracellular parasites are associated with Th1 responses. Although laboratory models have reported that Th1 and Th2 can be antagonistic, this has been challenged by studies of natural infections.

**Methods:**

Between 2019 and 2022 we completed 759 captures of 538 wild Soay sheep (1–4 captures per animal) and monitored body weight, parasite egg counts, Th phenotypes, cytokines, and GIN-specific antibodies.

**Results:**

While different Th cell counts, cytokines and antibody isotypes were generally positively correlated with each other, no strong positive associations were observed between these measurements. Cell counts had low repeatability (among-individual variation) across 4 years, while antibody levels were highly repeatable. The Th1 and Th2 cytokines Interferon-gamma (IFN-γ) and Interleukin-4 (IL-4) were moderately repeatable and were positively correlated at both the between- and within-individual levels independent of body condition or parasite exposure. IL-4 was negatively associated with GIN faecal egg count, while IFN-γ was negatively associated with coccidian faecal oocyst count, suggesting that these cytokines reflect resistance to these parasites. None of our immune markers were strongly associated with lamb survival.

**Conclusions:**

Our results provide insights into how different aspects of immune function interact to produce effective responses to complex infections but suggest longer-term data collection is required to address the causes of these interactions and to detect fitness consequences of variation in T cell phenotypes under natural conditions.

## Graphical Abstract

**Graphical Abstract:**
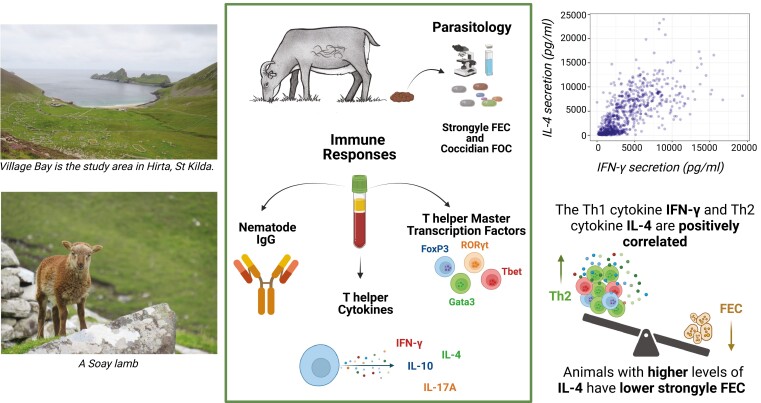

## Introduction

Most of our understanding of how the mammalian immune system works comes from studies in mouse models. The controlled, and even axenic environment that mouse models live in bears no resemblance to the natural conditions experienced by humans, livestock and particularly wildlife, where myriad biotic and abiotic factors can affect the ability of individuals to respond to infections. As such, extrapolating findings from laboratory immunology to the wild is challenging [1]. Furthermore, immune cell distribution, phenotypes and diversity in laboratory models are not necessarily shared by other mammalian species [2]. A growing body of work shows stark differences in the immune responses of laboratory rodents compared to their wild counterparts [1, 3, 4], illustrating the importance of studying how wild animals respond to infections [3, 5]. Unlike their laboratory counterparts, wild mammals experience diverse challenges ranging from co-infections to food limitations which will affect how the immune system responds to different challenges [2]. Understanding how and why the immune system varies amongst individuals in wild populations, and how different aspects of immunity are associated with parasite infection and fitness, could help us to determine how natural selection shapes immune responses. A major barrier to this goal is that measuring immune responses in wild animals is challenging due to logistical difficulties and restrictions on sample collection and processing in the field [4]. Furthermore, the difficulty of recapturing the same individuals and the paucity of immunological tools, makes many wild mammal populations unsuitable for such analyses. In recent years, however, advances in the availability of immunological tools for wild systems and the possibility of applying immune tools from domestic animals, have enabled insight into how immune responses in wild animals have been shaped [3, 6].

Immunological responses to infections are often a combination of innate and adaptive immunity. While the first is a non-specific type of response, adaptive immunity is more targeted to specific pathogens and as such will depend on the immunological experiences of the individual throughout their life. Different modes of response are required to tackle different infections: for instance, while responses to bacteria or viruses can target particular antigens on their surfaces to prevent cellular attachment and uptake, responses to metazoan parasites will mostly target different antigens, such as excretory/secretory products produced by nematodes to manipulate the host’s immune response and promote parasite survival [7]. The adaptive immune response is coordinated by cluster of differentiation 4 (CD4)^+^ T-helper (Th) cells which produce specific types of cytokines to shape the downstream effector immune response [8]. The initial paradigm of the Th1/Th2 differentiation of CD4^+^ Th cells by Mosmann and Coffman [9], was challenged with the discovery of Th17 cells [10], after which new Th subsets started to emerge [11, 12]. We now know that the polarization of the immune response by Th cells results in a plethora of different types of immune response, including Th1, Th2, Th17, and regulatory T cell (Treg) responses, which promote cell-mediated immunity, antibody-mediated immunity, mucosal/antibacterial immunity, and immune regulation, respectively [13]. Each of these subsets are characterized by the expression of associated master transcription factors which will direct the differentiation of naïve T cells to particular Th subsets and will be accompanied by the secretion of a specific cocktail of cytokines which will trigger signalling pathways. In laboratory mono-infection models, highly polarized responses have been shown to be driven by different pathogens [14], with Th1/Th2 responses being frequently described as antagonistic [15]. In more natural conditions, however, co-infections are the norm and consequently a mixed immune response will often be more advantageous. The specificity of this response is crucial, as if the immune response is not tailored appropriately to the infecting pathogens this can lead to persistent infections, immunopathology and decreased fitness [16]. In wild systems, a balanced immune response appears preferable for the survival of the individual: for example, African buffalo with a heightened Th2 response are more resistant to helminths but are unable to control infection with bovine Tuberculosis (BTB) which requires a Th1 response, leading to a quicker development of BTB and mortality [17]. Nematode infections not only generate Th2 responses, Tregs have been shown to be induced by the parasites themselves either by the secretion of specific factors or by interacting with local cell populations to attenuate the host’s inflammatory responses [18].

Before we can determine how natural selection has shaped immune response in the wild it is important to establish the extent to which individuals vary in their responses to infection (how repeatable immune responses are among individuals) and how heritable they are. Repeatability is the proportion of variance in a phenotype that is explained by between-individual differences [19] and has broadly two components: an additive genetic component and an environmental component. Heritability is the proportion of phenotypic variance explained by these additive genetic effects [20]; since this is a subset of the among-individual variation, repeatability is usually higher than heritability. Both are expressed on a scale of 0–1. Many studies have reported repeatable and heritable immune responses in wild populations, although these are often generic responses not linked to specific parasite infections [21, 22]. The genetics of antibody-mediated immunity to gastrointestinal nematodes (GIN) has been studied for decades in livestock and especially sheep because of its potential as a target for selective breeding for GIN resistance [23]. In contrast, studies of genetic variance in Th-mediated immunity are rare in any population. Cytokine responses in humans are underpinned by significant heritable variation [24, 25] and recent studies in cattle have found that the percentage of different CD4+ cell subsets are both moderately repeatable and heritable [26] and that total blood lymphocyte counts are heritable [27]. Meanwhile, a recent study suggested that cytokine responses in domestic sheep are heritable, with estimates ranging from 0.14 to 0.77 [28]. Furthermore, positive genetic correlations were identified between the production of the Th1-associated cytokine IFN-γ and the Th2-associated cytokine IL-4, and between the production of both IL-4 and IL-10 and circulating levels of GIN-specific IgA [28]. These results suggest that we should expect to see significant amounts of between-individual and genetic variation in Th immunity in the wild.

The wild Soay sheep population of St Kilda has been intensively studied since 1985 [29]. The Soays are infected with a variety of gastrointestinal parasites, the most prevalent and abundant of which are strongyle nematodes and apicomplexan coccidia [30], which would be predicted to be controlled by Th2 and Th1-type responses, respectively. Infections with both types of parasites are higher in lambs [30], which in part reflects both the naivety and immaturity of the immune response in early life [31]. Antibodies are a key effector of immunity to strongyles in sheep and are stimulated through a Th2-driven response [32]. We have measured these antibodies in ~30 years of blood samples and have found that they are highly repeatable and heritable [31] and associated with resistance to nematodes [33, 34] and survival [34, 35]. More recently we have explored the cell-mediated response, using flow cytometry to enumerate CD4^+^ cells expressing transcription factors associated with different Th phenotypes, and measuring cytokine secretion in whole blood stimulated with T cell mitogen. Our previous work, which analysed the first year of data incorporated here, suggested positive correlations among different Th cell counts and cytokine levels, but not between Th cell counts and cytokines. Furthermore, the production of IFN-γ was negatively associated with coccidian faecal oocysts counts (FOC) while the production of IL-4 was negatively associated with strongyle faecal egg counts (FEC), supporting our prediction that coccidian and strongyle parasites are controlled by Th1 and Th2-type immune responses, respectively [6]. In this study, we analysed a larger data set with 4 years of data on Th cell phenotypes and cytokines, with additional data on GIN-specific antibody responses. This 4-year data set enabled us to (i) validate our initial findings with more data; (ii) estimate the repeatability and heritability of cell-mediated immune traits; (iii) identify associations between cell-mediated and humoral immunity; (iv) estimate associations between cell-mediated immune traits and annual survival.

## Materials and methods

### Study population and data collection

Soay sheep have lived unmanaged in the St Kilda archipelago, NW Scotland (57°49ʹN, 08°34ʹW) for several thousand years. The human population of the island of Hirta (638 ha) was evacuated in 1930 and in 1934, 107 sheep were moved from the island of Soay (99 ha) onto Hirta [36]. The population was never managed and has grown, currently fluctuating between around 1300 and 2100 animals. Since 1985, the sheep living in the Village Bay area of Hirta (encompassing ~1/3 of the island and the sheep population) have been the subject of an intensive individual-based study. In the spring of each year, > 95% of the lambs born in Village Bay are captured within a week of birth, given identifying ear tags and weighed, blood-sampled and tissue-sampled for genotyping. In August, ~50% of the Village Bay population are captured and data are collected on weight, morphometrics, and blood and faecal samples are collected. The blood samples used in this study were collected from 538 individuals in August across 4 years (2019–2022). Natural mortality occurs in this population over the winter months (mainly January–March). Regular censusing of the population and mortality searching through late winter and spring means that carcases are recovered, and the approximate timing of death is known for most individuals.

### Blood collection

Blood collection was carried out by jugular venepuncture into lithium heparin vacutainers (Greiner Bio-One International GmbH). Due to time constraints during field sampling, samples were stored at 4˚C overnight prior to being processed, less than 24 h post capture.

Blood samples were used for the three main protocols detailed below: whole blood stimulations for cytokine secretion analysis, flow cytometry identification of CD4^+^ T cells expressing key Th master transcription factors and finally plasma was collected from the remainer of the sample for worm-specific antibody ELISAs.

### Whole blood stimulation assays

Whole blood stimulations were carried out by mixing equal volumes of whole blood and tissue culture media (RPMI-1640) supplemented with 10% Foetal Bovine Serum, 50 μM 2-mercaptoethanol, 2 mM l-glutamine, 100 U/ml Penicillin and 100 μg/ml Streptomycin, and 5 μg/ml Gentamicin (all from Sigma-Aldrich, UK). Media contained either 10 μg/ml of pokeweed mitogen (PWM, Sigma-Aldrich, UK) or the same volume of PBS (unstimulated control). Samples were incubated at 37˚C for 48 h and then centrifuged at 300 × *g* for 5 min. Supernatants were collected and stored at −20˚C until required for a period not longer than 3 months.

Secretion of the main cytokines representing the different Th subsets, namely interferon (IFN)-γ (Th1), interleukin (IL)-4 (Th2), IL-17A (Th17), and IL-10 (Treg), following whole stimulations were quantified by capture ELISA as detailed below. Quantification of ovine IL-4 and IFN-γ was carried out using commercial ELISA kits following the manufacturer’s instructions (MABTECH AB, Augustendalsvägen, SE, Sweden). Ovine IL-17A enzyme-linked immunosorbent assay (ELISA) was carried out using commercially available polyclonal rabbit anti-bovine IL-17A antibodies alongside bovine recombinant protein (Kingfisher Biotech, Inc., St. Paul, MN) and finally, mouse monoclonal anti-bovine IL-10 capture and detection antibodies (clones CC318 and CC320b, respectively, both from BioRad) were used alongside supernatants from COS-7 cells (African green monkey kidney fibroblast-like cell line that have been transformed by a defective SV40 virus) transfected with bovine IL-10 [37, 38] to measure IL-10 secretion. All incubations detailed below were carried out at room temperature unless stated and washing steps were performed six times with 350 μl PBST (Phosphate Buffered Saline [PBS] + 0.05% Tween 20) using a Thermo Scientific Wellwash™ Versa (ThermoFisher). Briefly, high-binding capacity ELISA plates (Immulon™ 2 HB 96-well plates, ThermoFisher) were incubated with their respective coating antibodies overnight at 4°C. Excess antibody was then washed off as detailed above and blocked for 1 h with blocking buffer (for IL-4, IFN-γ, and IL-17A: PBST plus 0.1% Bovine Serum Albumin (BSA, Sigma, UK) and for IL-10: PBS plus 3% of BSA). Following blocking step, plates were washed and 50 μl of supernatants, dilution buffer (negative control) or standards were added in duplicate for 1 h. A further wash was then carried out and detection antibodies were added for 1 h. Plates were washed once more and Streptavidin-horseradish peroxidase (HRP) (Dako, Agilent, Santa Clara, USA) was added to all the wells for 45 min. After the final washing step, SureBlue TMB (3,3′,5,5′-Tetramethylbenzidine) substrate (Insight Biotechnology, London, UK) was added and after 7–10 min the reaction was stopped by the addition of an equal volume of TMB stop solution (Insight Biotechnology, London, UK). Absorbance values were read at O.D. 450 nm. Samples were analysed at the following dilutions, 1:20 (IFN-γ), 1:4 (IL-4), neat (IL-17A), or 1:4 (IL-10). Cytokine concentrations were calculated using standard curves consisting of seven serial dilutions of recombinant cytokines ranging from 6.25 to 400 pg/ml for IFN-γ; 31.25 to 2000 pg/ml for IL-4; 23.43 to 1500 pg/ml for IL-17A and 0.206 to 13.2 Biological Units/ml for IL-10 [38].To account for any non-PWM-specific cytokine release, the concentration obtained from the control sample (media with PBS) was subtracted from that obtained from the PWM-stimulated samples, and the result was multiplied by the cytokine’s corresponding dilution factor.

### Flow cytometry analysis and leukocyte quantification

Multiple colour flow cytometric analysis was used to quantify CD4^+^ T cells expressing transcription factors representing different Th subsets (T-bet for Th1; GATA3 for Th2; RORγt for Th17; Foxp3 for Treg) as follows. Red blood cells were lysed from 2 ml of blood using warm red blood cell lysis buffer (1.5M NH_4_Cl, 100 mM NaHCO_3_, 10 mM N_2_ EDTA in ddH_2_O) and following a 2 min incubation, cells were washed twice with PBS. The resulting pellet was incubated with Zombie Violet™ Fixable dead cell stain (Biolegend, USA) for 15 min at RT in the dark. Excess dye was washed off with PBS and cells were stained with a mouse anti-ovine CD4 monoclonal antibody labelled to Alexa Fluor^®^ 647 (clone 44.38, IgG2a, BioRad) for 20 min at RT in the dark. Following two washes with FACS buffer (PBS + 5%FBS + 0.05%NaN_3_) cells were fixed with Foxp3 Staining Buffer Set buffer (Miltenyi Biotec, Bergisch Gladbach, Germany) for 30 min at 4˚C. After fixation, cells were washed twice with FACS buffer, re-suspended in 1 ml of PBS and stored at 4˚C for the remaining of the sampling period. The remaining of the staining protocol was carried out at Moredun Research Institute (MRI) within a month of sample collection. Briefly, cells were permeabilised and stained with monoclonal antibodies specific for Th-associated transcription factors: T-bet (clone REA102/4B10, Rec Human IgG1), GATA3 (clone REA174/TWAJ, Rec Human IgG1), RORγt (clone REA278/AFKJS-9, Rec Human IgG1), and Foxp3 (clone FJK-16s, Rat IgG2a, eBioscience) alongside Isotype control antibody (clone REA293). All antibodies were conjugated to phycoerythrin (PE) and were obtained from Miltenyi Biotech unless stated differently. Following incubation at 4°C for 30 min, cells were washed twice with permeabilization buffer and samples were analysed immediately. A minimum of 100 000 events were acquired using a Sony SA3800 Spectral Analyzer (Sony Biotechnology, Ltd) and analysed using FlowJo v10.8.1 for Windows 7.

The percentage of CD4^+^ and CD4^+^ T cells expressing each of the Th-associated transcription factors was calculated using the gating strategy described in [6] with an example shown in Supplementary Fig. S1. Briefly, initial gates were created to eliminate any dead cells and doublets by plotting the forward scatter height against area (FSC-H vs FSC-A) (Supplementary Fig. S1A and B). A gate was then created to include all the live, single white blood cells (leukocytes) present in the sample which include peripheral blood mononuclear cells (PBMC), granulocytes and neutrophils and inside of this, PBMC were then gated based on FSC-A and side scatter (SSC-A) (Supplementary Fig. S1C). CD4^+^ T cells (Supplementary Fig. S1D and E) and transcription factor positive CD4^+^ T cells (Supplementary Fig. S1F) were gated based on the fluorescence minus one control. Consequently, the data obtained represented the percentage of CD4^+^ T cells in PBMC and the percentage of CD4^+^ T cells expressing T-bet (Th1), GATA3 (Th2), RORγt (Th17), or Foxp3 (Treg) (Supplementary Fig. S1 G–J, respectively).

Data was then expressed as the percentage and the count per ml of blood of the total leukocyte population. Cell counts were used to calculate the total number of CD4^+^ T cells expressing each transcription factor per ml of blood and were carried out as follows. An aliquot of 20 μl of fresh heparinized blood was gently mixed with 180 μl of Solution 17 (ChemoMetec) and incubated for 10 min in a heat block at 37˚C. The sample was then gently mixed and loaded into a Via1-Cassette™ (ChemoMetec) prior to counting on a Nucleocounter NC-200 cell counter (ChemoMetec, Denmark). The total leukocyte counts per ml blood was then obtained and alongside the flow cytometry results used to calculate the total number of cells expressing each of the transcription factors.

### Parasite-specific antibody ELISA

Antibody ELISA was carried out to detect specific Immunoglobulin (Ig)G, IgA, and IgE levels against native *Teladorsagia circumcincta* (Tci) L3 somatic antigen from plasma (Tci-IgG, Tci-IgA, and Tci-IgE, respectively). All washes were carried out five times with Tris-buffered saline + 0.5% of Tween 20 (TBST) and incubations at 37°C for 1 h unless stated. High-binding capacity ELISA plates (Immulon™ 2 HB 96-well plates, ThermoFisher) were coated with 2 μg/ml of *T.circumcinta* L3 somatic antigen overnight at 4˚C. Plates were then washed three times with TBST and samples diluted 1:12 800 for Tci-IgG and 1:50 for Tci-IgA and Tci-IgE, positive control (pool of 20 serum samples from a combination of age/sex/sampling year), and negative control (TBST) added to the wells in duplicate. Following incubation, plates were washed and the following antibodies were added to all wells: rabbit anti-sheep IgG (H + L):HRP (BioRad), rabbit anti-sheep IgA:HRP (BioRad) and anti-sheep IgE (mouse monoclonal IgG1, clone 2F1, MRI). Since IgE antibody was not conjugated to HRP, following incubation and washing, goat polyclonal anti-mouse IgG1-HRP detection antibody (AbD Serotec STAR132P) was added and incubated a further 1h. Plates were then washed and SureBlue TMB substrate was added to all wells, incubated for 5–10 min and the reaction was stopped using an equal volume of 1M HCl. Absorbance values were read at O.D. 450 nm. ODs were blank corrected and to account for any inter-plate or any day-to-day variation, all results were normalized to the positive controls.

### Faecal egg and oocyst counts

The most prevalent parasites among Soay sheep on St Kilda are gastrointestinal strongyle nematodes (six species) and coccidia (11 *Eimeria* species) [30]. Faecal samples were collected rectally at August capture and stored anaerobically at 4˚C until processed within 2 weeks post collection. FEC for strongyle nematodes and faecal oocyst counts (FOC) for coccidia were carried out using a modified salt-flotation technique [39] described in [6].

### Statistical analysis

**Figure 1:**
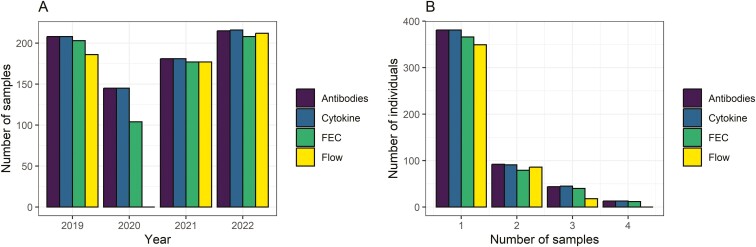
A breakdown of the number of samples of each type collected in each year (A) and the number of individual samples 1–4 times for each of the different phenotypes (B).

Seven hundred fifty-nine blood samples were collected from 538 individuals across 2019–2022. Nine samples were from individuals of uncertain age and were excluded from all analysis, leaving 750 samples from 530 individuals. All 750 samples were analysed for cytokine levels, but flow cytometry, antibody and FEC data were only available for a proportion of these samples (*N* = 575, 749 and 692, respectively); 2020 fieldwork was restricted by the Coronavirus disease (COVID)-19 pandemic and field preparation of samples for flow cytometry was not possible. Five hundred fifty-nine samples had complete data on cytokines, flow cytometry, antibodies, and FEC. A graphical representation of the data collected is shown in Fig. 1.

#### Correlations between immune variables

All statistical analyses were conducted in R version 4.3.2 [40]. First, the correlations among each of the 13 immunological variables were analysed for each year separately. To ensure that correlations were not explained by common age- and/or sex-related variation between variables, age and sex were first corrected for by fitting (generalized) linear models (GLMs) to data from each variable from each year, with explanatory variables of sex, age (an 11-level factor) and their interaction. For the six flow cytometry variables (PBMC, CD4^+^, CD4^+^T-bet^+^, CD4^+^GATA3^+^, CD4^+^RORγt^+^, and CD4^+^Foxp3^+^) negative binomial GLMs were fitted using the ‘MASS’ R package [41]; for IFN-γ, IL-17A, IL-10, Tci-IgG, Tci-IgA, and Tci-IgE linear models were fitted; and for IL-4 a linear model was fitted to natural log-transformed data. The residuals from these models were taken into a correlation analysis to quantify correlations after accounting for age and sex, estimating Spearman’s rank correlations among each pair of variables and assessing statistical significance using the ‘cor.mtest()’ function in the ‘corrplot’ R package [42].

#### Variation with age and sex

Next, variation in each of the immunological parameters with age and sex was analysed. Data sets of different sizes were used for flow cytometry traits (575 samples from 453 individuals), cytokines (750 samples, 530 individuals), and antibodies (746 samples, 527 individuals) due to missing data. For each of the 13 traits, 14 different (generalized) linear mixed-effects models were run describing age- and sex- related variation with age parameterized in different ways. The optimal error structure for each variable was determined by running models with different error structures for each of the variables (e.g. linear, Poisson, negative binomial, log-normal, and square-root transformed) and comparing models using the ‘DHARMa’ package [43]. The error structure used for each variable is given in Supplementary Table S1. All models included year (four levels) as a fixed effect and individual identity as a random effect. First, a null model (model 0) was fitted with just year and individual identity. Subsequent models were then fitted that added sex (model 1), age as linear, quadratic, a two-level factor (lambs versus other) and a four-level factor (lambs [aged under 1 year], yearlings [1 year old], adults [aged 2–6 years], geriatrics [aged > 6 years]) (models 2–5). Models 2–5 were then repeated, plus the main effect of sex (models 6–9) and with the interaction between age and sex (models 10-13). The 14 models for each trait were compared using Akaike’s Information Criterion (AIC) values, where the model with the lowest AIC was selected as best fitting the data, unless a simpler model had an AIC of ≤ 2 compared to the best model [44]. Once the best-fitting model had been established for each trait, significant variation with the year was tested by comparing the best-fitting model for each trait with a model that lacked the main effect of the year using likelihood ratio tests (LRTs). All models were run with the ‘glmmTMB’ R package [45].

#### Repeatability and heritability

Repeatability of strongyle FEC, coccidian FOC, and the 11 immunological variables were estimated, where repeatability is the proportion of phenotypic variance due to between-individual differences [19], using (generalized) linear mixed-effects models in the R package ‘MCMCglmm’ [46]. For FEC, FOC and the six flow cytometry variables, Poisson GLMMs were fitted and for the cytokine and antibody variables, LMMs were fitted with fixed effects of year (four levels), sex (two levels), and age category (lamb, yearling, adult, and geriatric), with a random effect of individual identity. Models were run for 110 000 iterations with a burn-in of 10 000 iterations and a thinning interval of 100 iterations for FEC, FOC, PBMC, CD4^+^ cells, IFN-γ, IL-4, IL-10, Tci-IgG, Tci-IgA, and Tci-IgE. The model was judged to have mixed effectively if the autocorrelation between estimates was < 0.1, if the effective sample size was ≥ 1000, and if the model passed Heidelberger and Welch’s convergence diagnostic using the ‘heidel.diag’ function from the ‘coda’ R package [47]. If the model did not successfully mix, the number of iterations, burn-in iterations and thinning interval were increased until the model mixed successfully. For CD4^+^T-bet^+^, CD4^+^GATA3^+^, CD4^+^RORγt^+^, CD4^+^Foxp3^+,^ and IL-17A, up to 620 000 iterations were used, with a burn-in of 20 000 iterations and a thinning interval of 200 iterations to obtain satisfactory mixing.

For those traits that had significant repeatability (see Results), the models were extended to quantitative genetic ‘animal models’ to estimate the heritability [48]. These models involve fitting the population pedigree [49] in the random effects structure of the model to partition the between-individual variance into ‘additive genetic’ and ‘permanent environment’ effects; the heritability is the proportion of phenotypic variance accounted for by additive genetic effects. The population pedigree was constructed following methods that have been previously described [49].

#### Association between IFN-γ and IL-4

The Th1 cytokine IFN-γ and the Th2 cytokine IL-4 were positively correlated, and both had significant repeatability (see Results). To explore the relationship between these two variables in more detail, a bivariate LMM was fitted including both IFN-γ and IL-4 as response variables, with individual- and residual-level variance-covariance matrices to estimate between- and within-individual correlations between the two traits. These models included the same fixed effects as the repeatability models described above. To determine whether these correlations could be driven by variation in condition or exposure to parasites, further models were fitted with body weight and/or strongyle FEC as additional fixed effects and the effect this had on the estimates of between- and within-individual covariation was determined.

#### Associations with strongyle FEC and coccidian FOC

To test whether our immune measures predicted parasite counts, negative binomial GLMMs of both strongyle FEC and coccidian FOC were fitted in ‘glmmTMB’ with the ‘nbinom2’ formulation. With either FEC or FOC as the response variable, a base model was fitted with fixed effects of age (two levels, lambs versus adults), sex, year, and the interaction between age and sex, plus a random effect of individual identity. Three additional models were fitted with each of the immunological variables as explanatory variables in turn: a model with the main effect of the immunological variable, a model with an interaction between the immune variable and age, and a model with the interaction between the immune variable and year. The relevant term in each model was tested with an LRT, comparing the model in question to the base model in the case of the main effect models, or comparing the interaction models to the main effect model. If any of these terms were supported for either FEC or FOC, all supported terms were fitted to the same model to test whether any associations between FEC/FOC and immune variables were supported independently of each other (i.e. having controlled/accounted for the effects of the other variables), again testing each term with LRTs.

#### Associations with lamb survival

Finally, predictors of winter survival were assessed in our data set. Analysis was restricted to lambs because there was a relatively even distribution of lambs surviving (*N* = 124) and dying (*N* = 149) over the winter, while only 9/264 adults sampled died over a subsequent winter. First-year survival was fitted as the response variable in a binomial GLM using the ‘glm’ function (0 = animal died before 31 May following the year of birth; 1 = animal survived past 31 May of the year following birth) with fixed effects of sex and year. Separate models were then fitted with weight, FEC, FOC, the immune variables as main effects, and interactions between these variables and year. Terms of interest were tested with LRTs as above. Any significant terms were taken forward into a model together to again test whether associations with survival were independent (i.e. present having controlled/accounted for the effects of the other variables).

## Results

### Correlations between immune variables

Our correlation analysis suggested that having corrected for age and sex, correlations among cytokine response levels, T-helper cell types, or *T. circumcincta*-specific antibodies were moderately positive and significant. In contrast, correlations *between* cytokine, T-helper cell type and antibodies were weaker and generally non-significant (Supplementary Fig. S2). These patterns were relatively consistent across years. The correlation between IFN-γ and IL-4 production was consistently high and positive across years (range 0.42–0.59; Supplementary Fig. S2).

### Variation with age and sex

The results of analyses of age and sex variation for each of the immunological variables are summarized in Supplementary Table S1 and Supplementary Figs. S3–S5. Among the Th cell count variables, the number of CD4^+^ cells was unrelated to age and sex; the number of PBMC and CD4^+^T-bet^+^ cells (Th1) was lower in adults than in lambs; the number of CD4^+^GATA3^+^ (Th2) and CD4^+^RORγt^+^ cells (Th17) was higher in females than males; and the number of CD4^+^Foxp3^+^ (Treg) cells declined in a linear fashion with age (Supplementary Fig. S3). The best-fitting model for each of the cytokine measures supported increases with age, while the increase was stronger for males than females for IL-17A and there was also a main effect of sex for IL-10 suggesting that males produced higher levels than females (Supplementary Fig. S4). Tci-IgG varied with age and sex: levels increased from lambs to yearlings, before declining in adults and geriatrics, though not to the very low levels of lambs, and females had higher average levels than males (Supplementary Fig. S5A). Meanwhile, Tci-IgA did not vary between the sexes but was higher in adults compared to lambs (Supplementary Fig. S5B), and Tci-IgE was higher in females compared to males and increased across the age classes (Supplementary Fig. S5C).

We included a year of sampling as a fixed effect in all models and found that all the immune traits varied significantly between years (LRT *P* < 0.001), apart from IL-4 where the year was marginally non-significant (*χ*^2^ = 7.43, Degrees of Freedom (DF) = 3, *P* = 0.059). This variation was inconsistent: among the Th cell phenotypes, PBMC, total CD4^+^ cells and CD4^+^Foxp3^+^ cells (Treg) were highest in 2022; CD4^+^GATA3^+^ (Th2) and CD4^+^RORγt^+^ cells (Th17) were highest in 2019 and CD4^+^T-bet^+^ (Th1) cells were highest in 2021 (Supplementary Fig. S6). Among cytokines, IFN-γ varied modestly with year but was highest in 2022; IL-4 did not vary with year; IL-17A and IL-10 both varied dramatically between years with IL-17A highest in 2019 and IL-10 highest in 2021 (Supplementary Fig. S7). Variation in the IL-17A data could be driven by some technical issues experienced with the different recombinant IL-17A standard batches used throughout the 4 years of the study, which produced protein concentration values significantly lower during 2021 and 2022 when compared to 2019–2020. However, when investigated further, not only were the patterns of cytokine secretion very similar between age groups/sexes in the different years, protein concentrations of original results of 2019–2020 and repeated ELISAs with the same samples, but using different standard batches were highly correlated to the original results (*r*^2^ = 0.95–0.97). Hence, the difference in the actual protein concentrations throughout our 4-year study should not have affected our analyses. Finally, all three *T. circumcincta*-specific antibodies varied modestly but significantly between years (Supplementary Fig. S8).

### Repeatability and heritability

Despite the relatively low proportion of individuals that were repeatedly sampled (Fig. 1), FEC, FOC, the number of CD4^+^ cells, and levels of IFN-γ, IL-4, Tci-IgG, Tci-IgA, and Tci-IgE returned non-zero repeatability estimates varying between 0.13 and 0.61 (Table 1). For the other traits, our models estimated between-individual variation to be bound by zero. This implies only those measures had any degree of consistent expression within individuals over our 4-year study (Supplementary Fig. S9). The *T. circumcincta*-specific antibodies were by far the most repeatable measure (0.52–0.61, implying > 50% of the variation was attributable to consistent among-individual differences in mean levels across years). IFN-γ, IL-4, and the number of CD4^+^ T cells had moderate repeatability (0.13–0.29), while FEC and FOC had relatively low repeatability (0.13).

**Table 1:** Variance components estimates from (generalized) linear mixed-effects models of immunological parameters. Individual and residual modes are from posterior distributions while repeatability is the proportion of phenotypic variance explained by individual identity. Repeatable immunological parameters are highlighted in bold. HPDI = highest posterior density interval

Trait	Individual mode (HPDI)	Residual mode	Repeatability (HPDI)
**FEC**	0.5187 (0.2798–0.7247)	0.9868 (0.8407–1.2875)	**0.13 (0.08–0.19)**
**FOC**	0.4998 (0.3461–0.7714)	1.0744 (0.9048–1.3106)	**0.13 (0.09–0.18)**
**PBMCs**	0.0002 (0.0000–0.0548)	0.1545 (0.1234–0.1941)	0.13 (0.00–0.26)
**CD4^+^**	0.0683 (0.0192–0.1157)	0.1739 (0.1439–0.2332)	**0.23 (0.07–0.38)**
**CD4^+^** **T-bet** **^+^**	0.0239 (0.0000–5.1076)	19.9762 (15.4456–24.3252)	0.00 (0.00–0.00)
**CD4^+^** **GATA3** **^+^**	0.0172 (0.0000–3.4994)	25.1969 (20.3383–28.8196)	0.00 (0.00–0.00)
**CD4^+^RORγt^+^**	0.0017 (0.0000–0.5848)	5.3843 (4.8235–6.4318)	0.00 (0.00–0.00)
**CD4^+^** **Foxp3** **^+^**	0.0023 (0.0000–0.5718)	5.551 (4.8883–6.4699)	0.00 (0.00–0.00)
**IFN-γ**	1 736 277 (1 068 509–2 328 000)	4 693 264 (4 108 511–5 514 061)	**0.28 (0.17–0.34)**
**IL-4**	2 025 520 (1 218 798–2 928 760)	5 390 963 (4 583 433–6 367 857)	**0.29 (0.17–0.37)**
**IL-17**	1 861 557 (5.8989–271 3290)	5 890 354 (497 065–8 035 426)	0.00 (0.00–0.34)
**IL-10**	10.1505 (0.0002–19.4371)	92.5611 (82.4753–109.4163)	0.01 (0.00–0.18)
**IgG**	0.0642 (0.0511–0.0777)	0.0421 (0.0350–0.0502)	**0.61 (0.52–0.68)**
**IgA**	0.1955 (0.1349–0.2462)	0.1716 (0.1461–0.2214)	**0.53 (0.39–0.62)**
**IgE**	0.0247 (0.0201–0.0295)	0.0179 (0.0152–0.0216)	**0.58 (0.50–0.64)**

We used the population pedigree to estimate the heritability of the eight traits that showed non-zero repeatability but found that in general the models mixed poorly: only the models of FEC, FOC and CD4^+^ cells mixed satisfactorily. CD4^+^ cells provided a heritability estimate of 0.18 (highest posterior density interval, HPDI = 0.07–0.31), while models of FEC and FOC provided heritability estimates of 0.09 (HPDI = 0.03–0.14) and 0.11 (HPDI = 0.06–0.16), respectively. The poor mixing of the other models was probably due to the relatively small sample size and the fact that few individuals were sampled more than once (Fig. 1), making it difficult for the model to separate additive genetic effects from other sources of between-individual variation.

### Association between IFN-γ and IL-4

**Figure 2:**
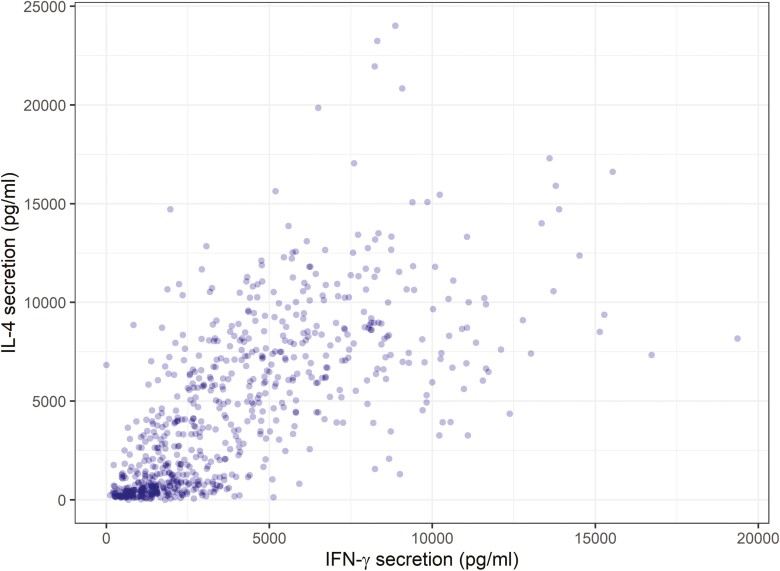
Raw cytokine secretion data showing the association between the Th1-associated cytokine IFN-y and the associated Th2-associated cytokine IL-4.

Bivariate linear mixed-effects models explored the degree to which the positive association between IFN-γ and IL-4 was driven by between- versus within-individual effects (Fig. 2). The base model, with fixed effects of age, sex, and year, supported a positive between-individual correlation (estimate = 0.34, HPDI = 0.04–0.64) and a positive within-individual (residual) correlation (estimate = 0.54, HPDI = 0.45–0.61). This implies that the positive association observed at the population level is due both to individuals having correlated mean levels of these cytokines across their lifetimes (between-individual) and having correlated year-to-year variation about those individuals' means (within-individual). These results did not change when fixed effects of strongyle FEC or body weight were added (Supplementary Table S2), suggesting the correlation is not driven by associated variation in body condition and parasite exposure jointly impacting both immune variables.

### Associations with strongyle FEC and coccidian FOC

**Figure 3:**
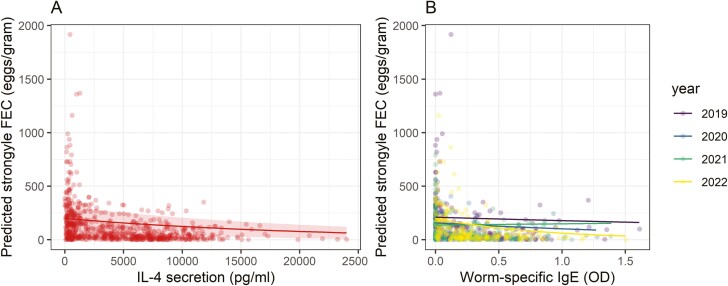
Associations between strongyle nematode FEC and immune variables. (A) Strongyle FEC was significantly negatively associated with IL-4 secretion, a relationship which held despite the presence of other immunological variables in the model. Meanwhile, FEC was also associated with (B) an interactive effect between year and T. circumcincta-specific IgE. Points show raw data; lines and shaded areas show model estimates ± 95%CI. Shaded areas are not shown in (B) in order to make the interactions easier to visualize.

Only three variables were significantly associated with strongyle FEC: the number of CD4^+^T-bet^+^ cells (Th1) in interaction with age class, IL-4 as a main effect and Tci-IgE in interaction with year (Supplementary Table S3). To test whether these effects were independent (i.e. present having controlled/accounted for other variables), we fitted a model of FEC with the age-by-CD4^+^T-bet^+^ interaction, the main effect of IL-4 and the year-by-Tci-IgE interaction, in a data set where all three variables were available (*N* = 559). In this model, the age-by-CD4^+^T-bet^+^ interaction was no longer supported (*χ*^2^ = 1.59, *P* = 0.127), but both the year-by-Tci-IgE interaction (*χ*^2^ = 9.99, *P* = 0.007) and the main effect of IL-4 were supported (*χ*^2^ = 7.68, *P* = 0.006). Given the age-by-CD4^+^T-bet^+^ interaction was not supported, we tested the IL-4 and Tci-IgE effects in a larger data set where we had information on both these variables (*N* = 692). Once again, the main effect of IL-4 was supported (*χ*^2^ = 10.67, *P* = 0.001), as was the year-by-Tci-IgE interaction (*χ*^2^ = 10.79, *P* = 0.012). IL-4 was negatively associated with FEC, suggesting a Th2-type response was associated with increased resistance to worms (Fig. 3A). The interaction between Tci-IgE and year suggested that the association between FEC and Tci-IgE varied between different years; specifically, there appeared to be weak associations in 2019 and 2021 and stronger, negative associations in 2020 and 2022 (Fig. 3B). To explore this further, we analysed the association between Tci-IgE and FEC in data from each year separately, accounting for IL-4, but in each case found no support for main effects of IgE (LRT, *P* > 0.05).

Three immunological variables were associated with coccidian FOC: IFN-γ, both as a main effect, and in interaction with year; IL-4, in interaction with year; and Tci-IgE, both as a main effect and in interaction with year (Supplementary Table S4). Once fitted in the same model, however, we found no support for either the main effect of IFN-γ (estimate = −0.1243, SE = 0.0661, *χ*^2^ = 3.49, *P* = 0.062) or the main effect of Tci-IgE (estimate = −0.1127, SE = 0.0655, *χ*^2^ = 2.92, *P* = 0.088), although both were suggestive of negative (resistance) effects. Similarly, the interactions between IFN-γ and year (*χ*^2^ = 3.68, *P* = 0.298), IL-4 and year (*χ*^2^ = 5.96, *P* = 0.113), and Tci-IgE and year (*χ*^2^ = 4.55, *P* = 0.208) were not supported when fitted in the same model. As such, we found no strong associations between coccidian FOC and our immunological variables.

### Associations with lamb survival

**Figure 4:**
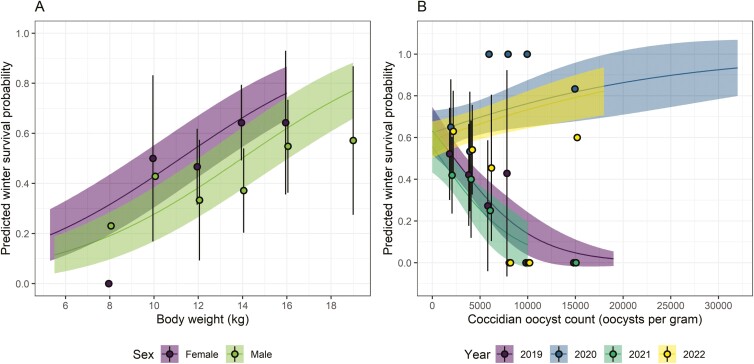
Generalised linear model results from Table S5 (right-hand side) showing significant predictors of lamb survival: (A) the main effects of sex and August body weight; (B) the interaction between coccidian FOC and year. Further analysis revealed the negative associations in 2019 and 2021 are significant, while the positive ones in 2020 and 2022 are not. Lines and shaded areas show model predictions ± 95% CI, while points and lines show mean survival in data pooled on the *x*-axis. For example, points corresponding to 8 kg on the *x*-axis of (A) shows mean survival in lambs weighing up to 8 kg; the point corresponding to 10 kg represents mean survival in lambs weighing 8–10 kg, and so on. In (B), data are sparse above 15 000 opg and so all values > 15 000 are pooled for each year. In both panels, predictions extend as far as the actual data on the *x*-axis.

We used binomial GLMMs to quantify associations between survival and immune parameters in lambs (*N* = 273). When fitting weight, FEC, FOC, and the immune parameters individually, we found that lamb survival was positively associated with body weight and Tci-IgE, negatively associated with strongyle FEC, and that there were FOC-by-year and CD4^+^GATA3^+^-by-year interactions (Supplementary Table S5). We next fitted all these terms in the same model in a reduced data set (*N* = 190), to account for missing 2020 data for CD4^+^GATA3^+^ (Th2) (Fig. 1). In this model (Table 2), strongyle FEC and Tci-IgE were no longer supported, nor was the interaction between CD4^+^GATA3^+^ and year. There was, however, still support for a main effect of sex, with females showing higher survival, and a main effect of weight, with heavier lambs being more likely to survive. There was also support for the interaction between coccidian FOC and year (Table 2). Since this data set used only 3 years of data due to the presence of CD4^+^GATA3^+^ in the model and given that CD4^+^GATA3^+^ was not significant, we then fitted weight, FEC, Tci-IgE and FOC-by-year into the same model using the full 4-year dataset (*N* = 242). Once again, FEC and Tci-IgE were not supported, and survival was higher in females and heavier lambs (Table 2, Fig. 4A), and the interaction between FOC and year was supported (Table 2). We explored the interaction further by analysing the association between FOC and survival in each of the 4 years separately. FOC was negatively associated with survival in 2019 (estimate = −0.0003, Standard Error (SE) = 0.0001, *χ*^2^ = 8.07, *P* = 0.005) and 2021 (estimate = −0.0003, SE = 0.0002, *χ*^2^ = 5.39, *P* = 0.020), but was not associated with survival in 2020 (estimate = 0.0001, SE = 0.0001, *χ*^2^ = 2.05, *P* = 0.152) or 2022 (estimate = 0.0000, SE = 0.0001, *χ*^2^ = 0.39, *P* = 0.531). In summary, coccidian FOC was negatively associated with survival in some years but not others (Fig. 4B).

**Table 2:** Full results of models of lamb first winter survival. On the left, a model including the interaction between CD4 + GATA3 + cells (Th2) and year run on a small data set of 199 lambs. On the right, the model was re-run on a larger data set, excluding the CD4 + GATA3 + interaction (*N* = 242), and in both cases, there is support for the main effects of weight and sex, and for the interaction between coccidian FOC and year

	Data with CD4 + GATA3 + (Th2) (*N* = 190)			Data without CD4 + GATA3 + (Th2) (*N* = 242)		
Variable	Estimate (SE)	χ² (DF)	*P*	Estimate (SE)	*χ*² (DF)	*P*
Intercept	−2.0450 (1.2000)			−2.3070 (1.0440)		
Sex (Female)	0.0000 (0.0000)	6.1 (1)	0.014	0.0000 (0.0000)	4.58 (1)	0.032
Sex (Male)	−0.8587 (0.3549)			−0.6700 (0.3174)		
Body weight	0.2313 (0.0776)	9.82 (1)	0.002	0.2413 (0.0694)	13.38 (1)	<0.001
Year (2019)	0.0000 (0.0000)	NA	NA	0.0000 (0.0000)	NA	NA
Year (2020)	NA			−0.0393 (0.6236)		
Year (2021)	−0.5143 (0.7673)			−0.3164 (0.6661)		
Year (2022)	−0.0107 (0.6949)			−0.1750 (0.5942)		
FOC	−0.0002 (0.0001)	NA	NA	−0.0002 (0.0001)	NA	NA
FEC	−0.0007 (0.0008)	0.83 (1)	0.362	−0.0008 (0.0007)	1.43 (1)	0.230
IgE	1.6200 (3.6870)	0.19 (1)	0.66	3.0260 (3.2400)	0.89 (1)	0.349
CD4 + GATA3+	−2.1E-06 (4.3E-06)	NA	NA	NA (NA)	NA	NA
Year(2019): FOC	0.0000 (0.0000)	8.58 (2)	0.014	0.0000 (0.0000)	13.19 (1)	0.004
Year (2020): FOC	NA			0.0003 (0.0001)		
Year (2021): FOC	−0.0001 (0.0002)			−0.0000 (0.0002)		
Year (2022): FOC	0.0003 (0.0001)			0.0003 (0.0001)		
Year(2019): CD4 + GATA3+	0.0000 (0.0000)	4.05 (2)	0.132	NA	NA	NA
Year (2021): CD4 + GATA3+	7.8E-06 (6.6E-06)			NA		
Year (2022): CD4 + GATA3+	02.1E-06 (5.7E-06)			NA		

## Discussion

Understanding the drivers of immunological variation in populations experiencing complex co-infections and challenging natural environments is important to establish how our knowledge of immunology generalizes beyond the laboratory [1, 5]. Our study represents one of the most comprehensive assessments of different markers of immune function and their relationships with parasite burdens and survival ever conducted in a wild animal population. The 4-year study shows that many patterns observed in a previous single-year analysis of Th immunity in the Soay sheep [6] are stable over time. We stimulated blood leukocytes with the polyclonal mitogen PWM to measure the capacity of T and B cells to proliferate in response to a non-specific challenge. This stimulant is commonly used as a proxy for immune system health, and in particular that of adaptive immunity [28, 50, 51], and has been shown to potentially be used as a tool for genetic selection for particular immune traits [28]. In our 4-year dataset, we found that following induction by PWM*, ex vivo* cytokine release across Th subsets increases with age and is positively correlated across subsets, with an especially strong and consistent positive relationship between Th1 and Th2 measures [6]. Although previous studies have shown an age-related shift from Th1 to Th2 responses [52], we did not detect such a shift in our data. However, a meta-analysis using more than 60 studies carried out in humans [52] found an inconsistency across studies which proved that this shift is not always present, and highlighted the importance of the stimuli used for the induction of cytokine secretion when carrying out such studies [53].

Using our 4-year data, we also analysed worm-specific antibody responses and demonstrated that these measures are uncorrelated with Th phenotypes. Consistent with our previous work, we found that higher levels of the Th2 cytokine IL-4 predicted reduced nematode FEC. Worm-resistant domestic sheep have an upregulated IL-4 response when compared to susceptible sheep [54] and similarly, IL-13 and IL-5, two other Th2-associated cytokines, have been negatively associated with worm infections in humans [55]. The negative IFN-γ–FOC relationship we observed previously [6] was also detected but was marginally non-significant in the 4-year data set. Using our longitudinal data, we also provide rare evidence from the wild that Th1 and Th2 cytokines are repeatable within individuals over time, and recapitulate previous work showing that antibody levels are highly repeatable in this system [31]. IFN-γ secretion following stimulation with PWM is heritable in blackface sheep [28], but our relatively small sample size limited our ability to test for heritable variation. We also tested whether the T-helper or humoral immune measures recorded in the summer predicted lamb mortality, as in our previous larger-scale studies we have shown that worm-specific IgG in the Soay sheep population is robustly and positively associated with adult mortality [34, 35]. However, in this study, we found no evidence for any associations with mortality, perhaps once more due to our limited sample size. Overall, our results provide new insights into how markers of cell-mediated and humoral immunity are related and vary across the lifetimes of individuals, whilst also highlighting the challenges of testing the heritability and health consequences of immunological variation in natural systems.

Our work adds to a growing literature on wild birds and mammals showing that some immune measures are repeatable across the lives of individuals [56]. Establishing repeatability is an important first step towards understanding the drivers of immune variation including additive genetic effects, maternal effects and other early-life influences, and environmental effects. We found that two key measures of Th1 and Th2 immunity, production of IFN-γ and IL-4 by circulating leukocytes respectively, are moderately repeatable over 4 years and also reiterates the finding that anti-worm antibody levels are moderately to strongly repeatable [31]. Although we attempted to determine the role of genetics using pedigree-based ‘animal models’, these models did not mix successfully, probably because of the relatively small sample size and a lack of statistical power. A previous analysis of anti-worm antibody levels in the Soay sheep, using a much larger sample size (*N* = 6543) showed that repeatable variation in Tci-IgG, Tci-IgA, and Tci-IgE was largely explained by additive genetic effects [31]. Meanwhile, a recent study of domestic sheep lambs with a sample size of > 1000, found that IL-4, IL-10, and IFN-γ cytokine release were all moderately to strongly heritable [28] and in humans PWM-induced IFN-γ release from whole blood was also heritable (*h*^2^ ~0.47 [57]). Taken together, these results suggest that a longer-term study of Th immune measures is required on St Kilda to estimate heritability reliably and reflects the challenge of collecting data in the field. Such challenges are common to estimating heritability for any trait in the wild [58], but immunological traits are far more difficult to measure than life-history and morphological traits. Recent studies in wild rodents and buffalo have suggested that early-life immune expression and more stable immune profiles may predict later infection status and reproductive performance [56, 59]. Testing both early-life environment effects on later immune phenotypes and the hypothesis that immune stability, rather than average immune state, may predict health outcomes remain important challenges, but would demand multi-decade immunological field studies in the Soay sheep population.

Laboratory studies have shown us that different T-helper cells are essential for coordinating different kinds of adaptive immune responses and suggest a commitment to Th1 and Th2 responses may be constrained such that either one or other predominates. And what is more, if an inappropriate response is chosen, this could lead to either persistent uncontrolled infections or immunopathology [60]. Despite this, there is now a growing body of evidence that under more natural co-infection conditions, Th1 and Th2 responses are positively correlated and vary in a way that does not suggest constraint. Gene expression of GATA3 and IFN-γ in wild rodents were positively correlated [61], while laboratory mice experiencing co-infections had positively correlated levels of IgG1 (Th2) and IgG2 (Th1) [62]. Further to these, in wildhouse mice on the Isle of May, Scotland, gene expression and circulating cytokines analysis showed positive correlations between Th1 and Th2 genes/proteins within the same tissue [63]. We have previously shown, using only 1 year of immune data from Soay sheep, that IFN-γ and IL-4 responses were positively correlated; here, we found that this positive correlation is present in 4 years, and that it is driven by both among- and within-individual associations. Broadly, the among-individual correlation suggests that individuals with high average IFN-γ responses should also have high average IL-4 responses. As with repeatability, this could be due to joint genetic or early environmental influences on the tendency to produce robust Th1 and Th2 responses. A recent study of domestic sheep found a strong positive genetic correlation between IFN-γ and IL-4 [28], supporting the notion that the association in our population is likely to have a genetic basis.

Two explanations that have been offered for mounting evidence of positive correlations among markers of commitment to different Th sub-types are among-individual heterogeneity in resource availability and joint exposure to different infections. Concerning the first of these, wild animals experience varying environmental conditions including food availability, which is often limited. Life-history theory states that individuals have a limited pool of resources which they must allocate between competing functions such as foraging, fighting infection, searching for mates, and investing in offspring or, more fundamentally, survival and reproduction, leading to the expectation that trade-offs should exist between these traits [64, 65]. However, expected trade-offs are often not observed, and indeed a more common result is for positive, rather than negative, associations between life-history traits [66–69]. Thus, the individuals with access to the most resources may be able to invest more in immune responses, impacting different aspects of immune function in a consistent way and creating a positive association between IL-4 and IFN-γ. Our analyses, however, control for variation in body mass in summer, which will reflect—to some degree—differences in resource acquisition. We also find that while Th1 and Th2 responses are positively correlated, they are not as strongly correlated with Th17 or Treg responses or with antibody levels. Both observations argue against resource availability being the driver of the positive IL-4–IFN-γ association here. However, if exposure to different infections is correlated over space or time, different arms of the immune system geared to dealing with different pathogens and parasites (e.g. Th1/Th2) might be jointly stimulated. Indeed, given the growing understanding of how spatially regulated the immune response is within the host [70], it is conceptually easy to imagine that different Th responses may be generated within the same host even if they are known to counter-regulate each other *in vitro*. Furthermore, it would be advantageous from a fitness perspective to be able to resist or tolerate more than one type of infection at the same time and natural selection may favour the evolution of a multi-pronged immune response. There is good evidence that exposure to different infections is correlated in unmanaged populations [71]. We observe strong positive correlations between FEC and FOC in Soay sheep [30, 72] which suggests potentially shared patterns of exposure. However, including FEC in our model of IFN-γ and IL-4 did not account for the observed positive correlation. Further work is required to better understand the degree to which joint exposure in space and time versus the lasting effects of genes and early environment on immune phenotypes are responsible for correlations among immune phenotypes under natural conditions.

How different aspects of immune function interact to produce coordinated and effective responses to complex, dynamic infection pressures remains an important open question. Long-term studies of wild vertebrates potentially represent important models to understand how immune variation predicts infection, health, and survival when co-infections predominate [73]. For example, studies of wild African buffalo in the Kruger National Park have explored the interplay between different Th responses as bovine tuberculosis (BTB) and GIN, which are controlled by Th1 and Th2 responses respectively, are endemic in this population [17]. These studies show potential trade-offs between Th1 and Th2 immunity, with BTB disease progression more rapid in worm-resistant animals, possibly due to lower Th1 responses [17]. A further study of this population has shown that reduced BTB mortality in buffalo where parasitic worms have been cleared via a long-acting anthelmintic bolus resulted in enhanced Th1 responses [74]. Clearly, these studies focus around Th1/Th2 counter-regulation and trade-offs, which may be more evident in populations where a high morbidity/mortality Th1-controlled pathogen such as BTB is endemic. In the St Kilda Soay sheep population, high mortality pathogens similar to those found in Kruger are not present [75] which may explain why we do not see obvious negative consequences of high Th2 responses in this population and in general we see positive correlations between Th1 and Th2 immunity. Clearly, both study systems contribute to our knowledge of the consequences of immune variation and point to significant environmental factors which will influence both the immune response and infection outcome.

Despite previous work linking anti-worm IgG and survival in adult sheep on St Kilda [35], here we found no evidence for associations between immune measures and lamb survival. Generally, estimates of associations between Th responses and fitness parameters such as survival is lacking in the wild, no doubt due to the difficulty of collecting data on both these parameters. In our study, this seems likely to reflect the fact that we were limited in our sample size for this analysis because lambs were the only age group that experienced sufficient mortality. Lambs are immunologically naïve and at the point we measure them aged around 4 months, their antibody and Th phenotypes may reflect a highly variable transition phase towards the relatively effective defence they acquire against infection by their second summer [30, 31]. It may be that relationships between immune phenotypes and survival are hard to detect during this development phase, and a focus on older animals would be required to reveal this. Certainly, all previous studies in this population that have detected relationships between antibody measures and measures of fitness and have found associations in adults but not lambs, have had much larger sample sizes, and have also often focussed on particularly high-mortality years [33–35]. Thus, a longer-term study of Th parameters in the Soay sheep with more sampling of adults may be required to reveal their associations with fitness.

The presence of a year-dependent relationship between protozoan FOC and lamb survival was novel and notable in our analyses. Previous studies have not linked FOC and mortality, but coccidiosis can cause severe pathology and even mortality in domestic lambs [76]. This suggests that the impact of coccidian parasites on lambs in St Kilda may be environment-dependent and potentially linked to climate conditions and the height of the peak in coccidian exposure, which would affect the pathological damage caused by high burdens to lambs in early spring. We should point out that faecal egg/oocyst counts are carried out in August, likely missing the peak of FOC, but high burdens are still observed in the summer in lambs. The impact of immunological variation in parasite burdens and health/fitness is likely complicated in wild systems, a point illustrated powerfully by a recent study of buffalo showing that Th immune measures related variably to worm and coccidian burdens depending on context (anthelmintic treatment or control) and also depended on the worm species involved [56].

In summary, we found that in wild Soay sheep, immunological markers of the same category were generally positively correlated, regardless of the type of Th response they are associated with. Several of these markers, most notably IFN-γ and IL-4, varied substantially between individuals, suggesting they are governed by factors such as genetics or early-life events. The positive association between IFN-γ and IL-4 was not explained by host body weight or FEC, suggesting a role for factors other than condition or exposure in driving this positive correlation. Higher IL-4 levels were associated with lower nematode FEC, suggesting IL-4 is associated with resistance to these worms, but we found no link between IL-4 and the humoral response to worms, which previous studies in this population suggest are important. Meanwhile, our limited ability to assess heritability and associations between immune markers, parasites, and survival, is likely linked to our relatively limited sample size, despite 4 years of intensive field and laboratory work. This limited power highlights the challenge field immunologists face, but our findings suggest that continuous monitoring of such traits in this population has the potential to enable a deeper understanding of the causes and consequences of immunological variation in wild populations [3, 73].

## Supplementary Material

kyae017_suppl_Supplementary_Figures_S1-S9_Tables_S1-S5

## Data Availability

All data used in this manuscript will be made available online upon final acceptance of the manuscript.
