# Supplementary material for: T-helper cell phenotypes are repeatable, positively correlated, and associated with helminth infection in wild Soay sheep
Source: Discov Immunol. 2025 Feb 8;4(1):kyae017. doi: 10.1093/discim/kyae017 (PMC11832277; doi:10.1093/discim/kyae017)
Supplement: kyae017_suppl_Supplementary_Figures_S1-S9_Tables_S1-S5 [file kyae017_suppl_supplementary_figures_s1-s9_tables_s1-s5.docx]

Supporting information for:

***T-helper cell phenotypes are repeatable, positively correlated and associated with helminth infection in wild Soay sheep***

Yolanda Corripio-Miyar, Adam D. Hayward, Hannah Lemon, Xavier Bal, Jill G. Pilkington, Josephine M. Pemberton, Daniel H. Nussey & Tom N. McNeilly


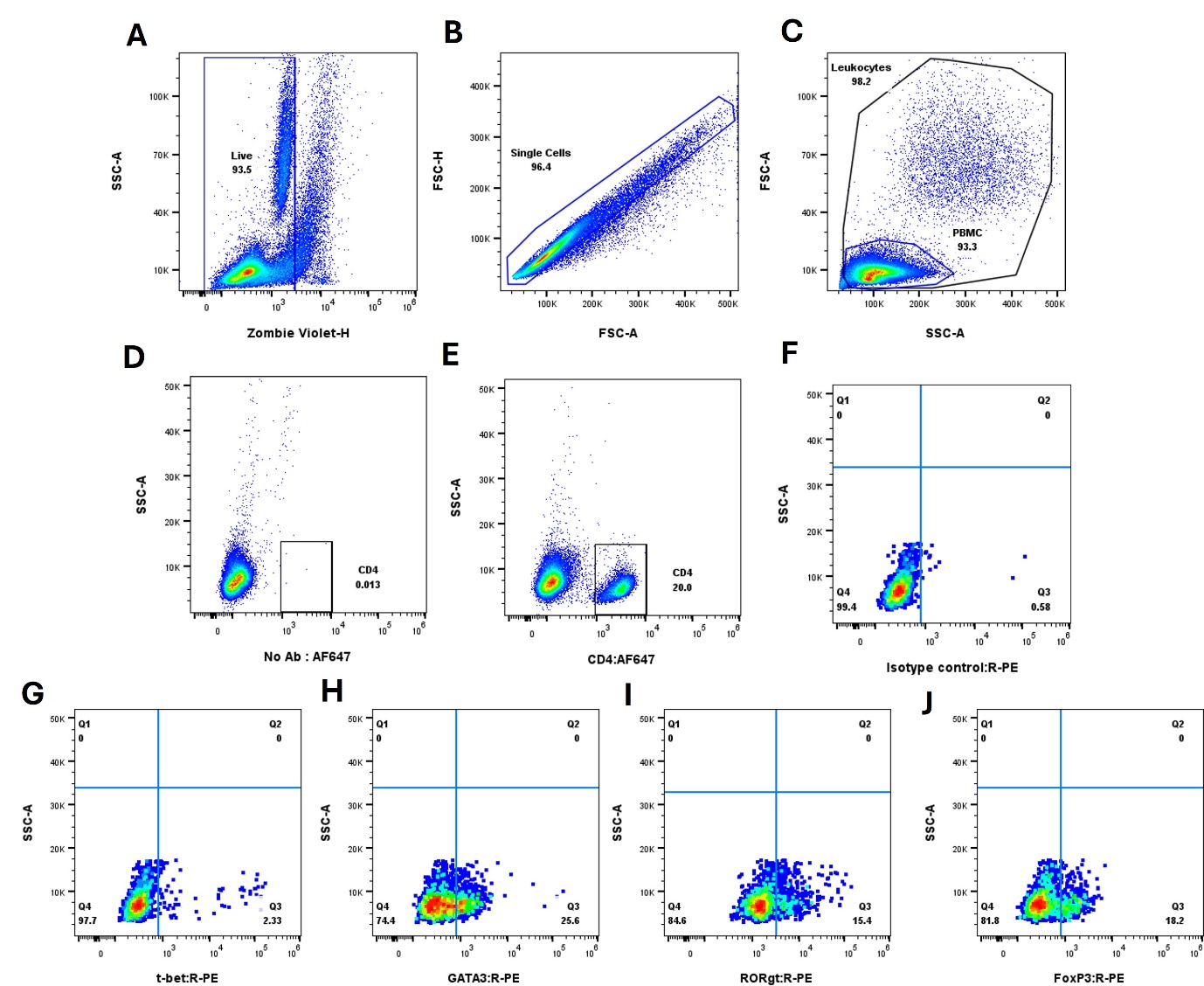


***Figure S1. Gating strategy for flow cytometry analysis.*** *Multicolour flow cytometry was used to determine the percentage of CD4 T cells expressing key Th transcription factors. Initially, cells were gated to eliminate dead cells (A) and single cells (B). A gate for PBMC (C) was then created guided by the FCS-A and SSC-A as part of the larger leukocyte gate. Gates for fluorochromes were set using the FMO controls as follows. The gate for CD4 conjugated to Alexa Fluor® 647 (E) was created following the no antibody control (D) gate. Similarly, gates set using the FMO control for R-PE (F) were used to determine the percentage of CD4 T cells expressing each of the Th transcription factors: T-bet (G, Th1), Gata3 (H, Th2), RORγt (I, Th17) and Foxp3 (J, Treg). A minimum of 100,000 events were acquired where enough cells were available.*


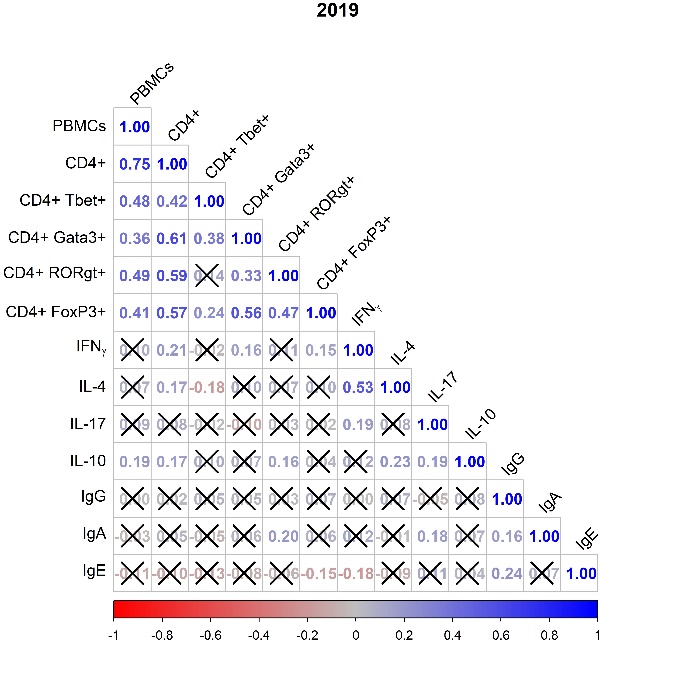

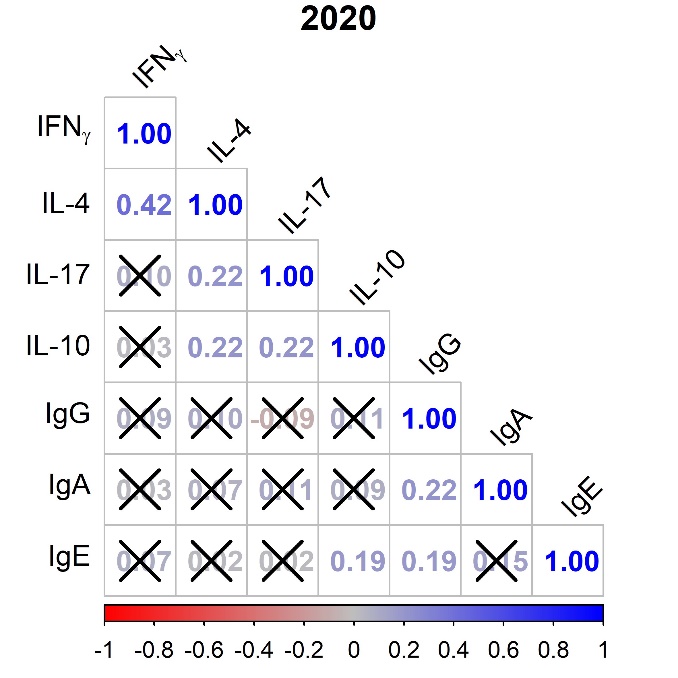


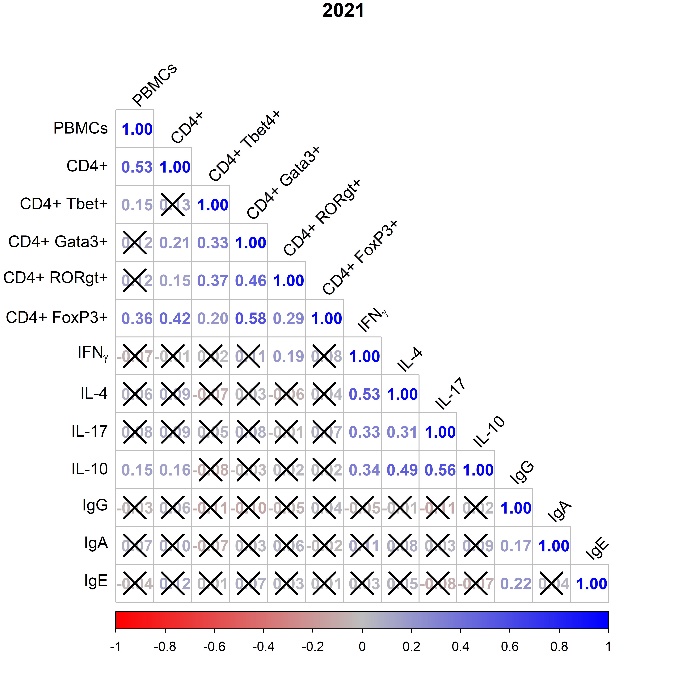

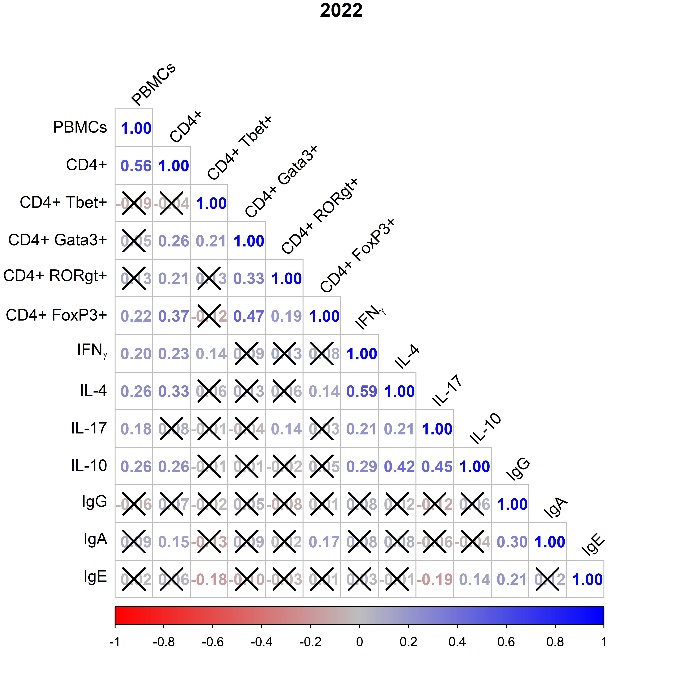


***Figure S2.*** *Correlation matrices showing Spearman’s rank correlations between pairs of immunological variables corrected for age and sex, where blue values indicate positive associations and red values indicate negative associations. Cell phenotypes represent cell counts per ml blood. Correlations with black cross were not statistically significant at α = 0.05. The different arms of the cell-mediated response represented are Th1 (CD4^+^T-bet^+^, IFN_γ_), Th2 (CD4^+^GATA3^+^, IL-4), Th17 (CD4^+^RORgt^+^, IL-17) and Treg (CD4^+^Foxp3^+^, IL-10).*

| **Number** | **Description** | **PBMC** | **CD4^+^** | **CD4^+^ T-bet^+^**  **(Th1)** | **CD4^+^ GATA3^+^**  **(Th2)** | **CD4^+^ RORγt^+^**  **(Th17)** | **CD4^+^ Foxp3^+^**  **(Treg)** | **IFN-γ**  **(Th1)** | **IL-4**  **(Th2)** | **IL-17A**  **(Th17)** | **IL-10**  **(Treg)** | **IgG** | **IgA** | **IgE** |
| --- | --- | --- | --- | --- | --- | --- | --- | --- | --- | --- | --- | --- | --- | --- |
| **Model** |  | NB | NB | ZINB | ZINB | ZINB | ZINB | SQRT | SQRT | SQRT | SQRT | Normal | Normal | Normal |
| **0** | Null | 17.32 | **0.35** | 10.78 | 2.85 | 7.98 | 9.43 | 335.39 | 792.33 | 124.32 | 151.35 | 545.08 | 208.12 | 466.34 |
| **1** | Sex | 19.28 | 1.01 | 13.00 | **0.93** | **0.86** | 7.43 | 326.35 | 777.30 | 125.61 | 152.90 | 502.89 | 192.75 | 456.57 |
| **2** | Age | 8.33 | 2.28 | 4.67 | 3.63 | 8.90 | **0.00** | 58.80 | 306.12 | 51.77 | 49.25 | 458.54 | 106.92 | 188.98 |
| **3** | Age² | 3.64 | **0.00** | **0.00** | 1.91 | 10.89 | 1.84 | 8.63 | 120.94 | 26.70 | 33.76 | 337.41 | 44.03 | 38.16 |
| **4** | Age(2) | **0.00** | 1.83 | **0.45** | NA | 10.14 | 6.29 | 76.57 | 97.79 | 31.86 | 29.76 | 61.85 | **0.36** | 50.52 |
| **5** | Age(4) | 1.75 | 0.54 | 3.27 | 9.23 | 15.35 | 4.90 | **0.00** | **1.53** | 26.21 | 17.65 | 17.28 | 1.02 | 4.15 |
| **6** | Sex + Age | 9.71 | 3.00 | 8.33 | 2.05 | 0.59 | 0.02 | 59.62 | 306.72 | 40.71 | 34.50 | 439.95 | 106.03 | 190.56 |
| **7** | Sex + Age² | 4.95 | 0.61 | 3.69 | NA | 2.24 | 1.79 | 9.16 | 120.68 | 14.88 | 17.91 | 315.25 | 43.07 | 39.60 |
| **8** | Sex + Age(4) | 1.28 | 1.96 | 4.19 | 2.01 | **0.00** | 4.74 | 78.56 | 99.64 | 22.89 | 18.28 | 52.60 | **0.00** | 52.52 |
| **9** | Sex + Age(2) | 2.69 | 0.57 | 7.13 | 8.52 | 4.75 | 4.34 | 0.64 | **0.00** | 13.74 | **0.00** | **0.00** | 0.84 | 5.15 |
| **10** | Sex*Age | 10.02 | 2.63 | 11.30 | **0.00** | 4.12 | 3.72 | 54.20 | 285.65 | 8.18 | 29.46 | 428.09 | 89.97 | 155.26 |
| **11** | Sex*Age² | 6.91 | 2.07 | 6.73 | 2.22 | 6.03 | 4.77 | 11.13 | 122.38 | **0.00** | 18.96 | 316.78 | 43.47 | 38.30 |
| **12** | Sex*Age(2) | 2.81 | 2.78 | 8.07 | 4.03 | 3.35 | 8.46 | 76.00 | 99.95 | 23.01 | 20.09 | 52.79 | 1.13 | 52.46 |
| **13** | Sex*Age(4) | 7.05 | 2.01 | 16.37 | 12.86 | 12.42 | 14.72 | 0.90 | 4.21 | 13.81 | 3.87 | 1.55 | 4.17 | **0.00** |

***Table S1.*** *A Comparison of models testing for age- and sex-specific variation in 13 different immunological variables. All models include year and individual identity as fixed and random effects respectively, and the terms listed under “Description”. “Age(2)” refers to age as a two-level categorical variable (lambs versus other animals) and “Age(4)” refers to age as a four-level categorical variable (lambs, yearlings, adults, geriatrics). The different parameterizations of age are described in the main text. Each column presents the ΔAIC values for the 14 models run for each of the immunological variables, relative to the model with the lowest AIC, which has ΔAIC = 0. The models highlighted in blue are plotted in Figure S2 (Th cell counts), S3 (cytokines) or S4 (*T. circumcincta*-specific antibodies) respectively and are either the model with the lowest AIC, or a simpler model with ΔAIC ≤ 2. The model highlighted in grey is the model with the lowest AIC, if it’s not the model that was plotted. NB = model was fitted with negative binomial errors; ZINB = zero-inflated negative binomial model; SQRT = data square-root transformed prior to analyses with linear mixed-effects model; Normal = linear mixed effects model on untransformed data.*


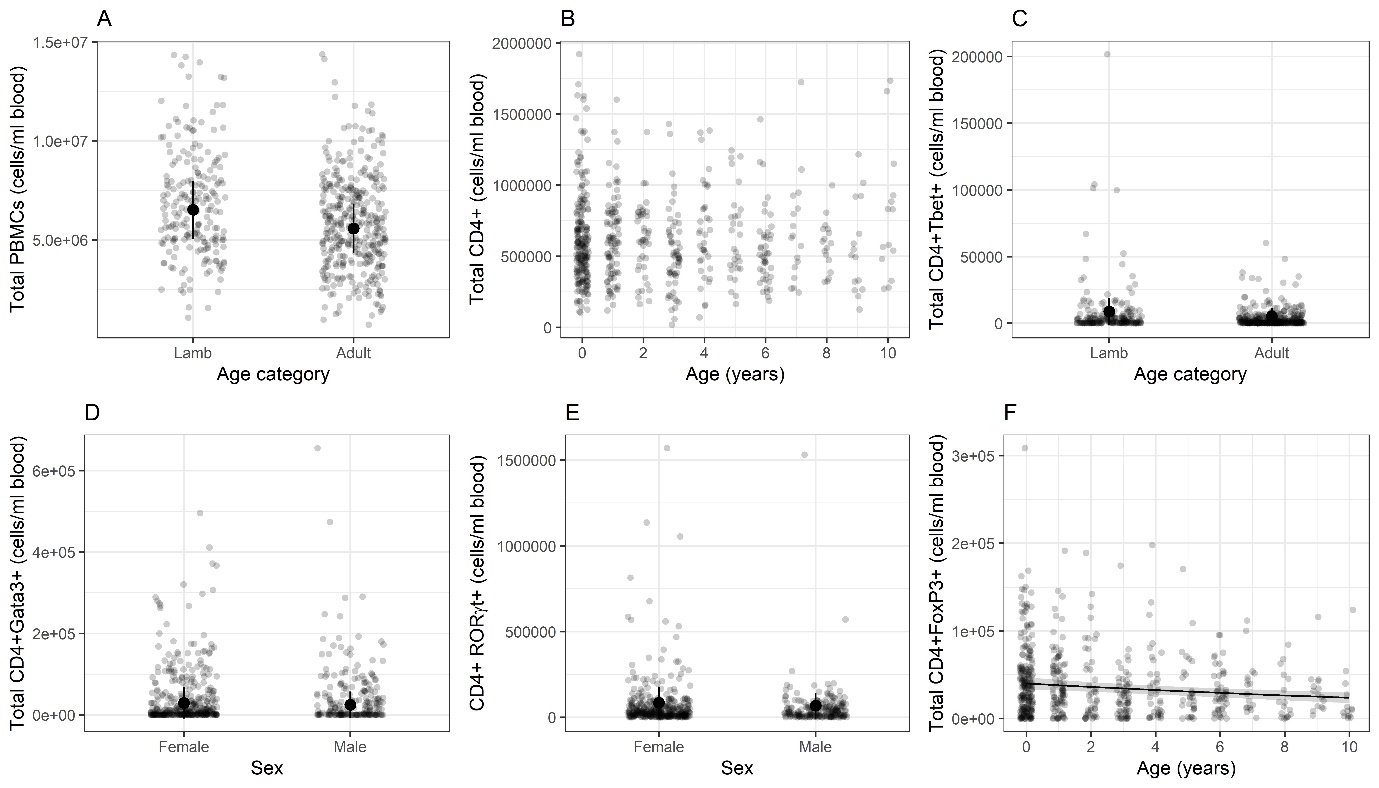


***Figure S3.*** *Age- and/or sex-specific variation in Th cell counts: (A) PBMC; (B) CD4+*

*cells; (C) CD4+T-bet+ cells (Th1); (D) CD4+GATA3+ cells (Th2); (E) CD4+RORγt+ cells (Th17); (F) CD4+Foxp3+ cells (Treg). Points show raw data; large points with bars show estimates ± 95% CI from model where age or sex was fitted as a factor and lines with shaded areas in (F) show estimates ± 95% CI from models where age was fitted as a continuous variable. For model details, see Supplementary Table S1.*


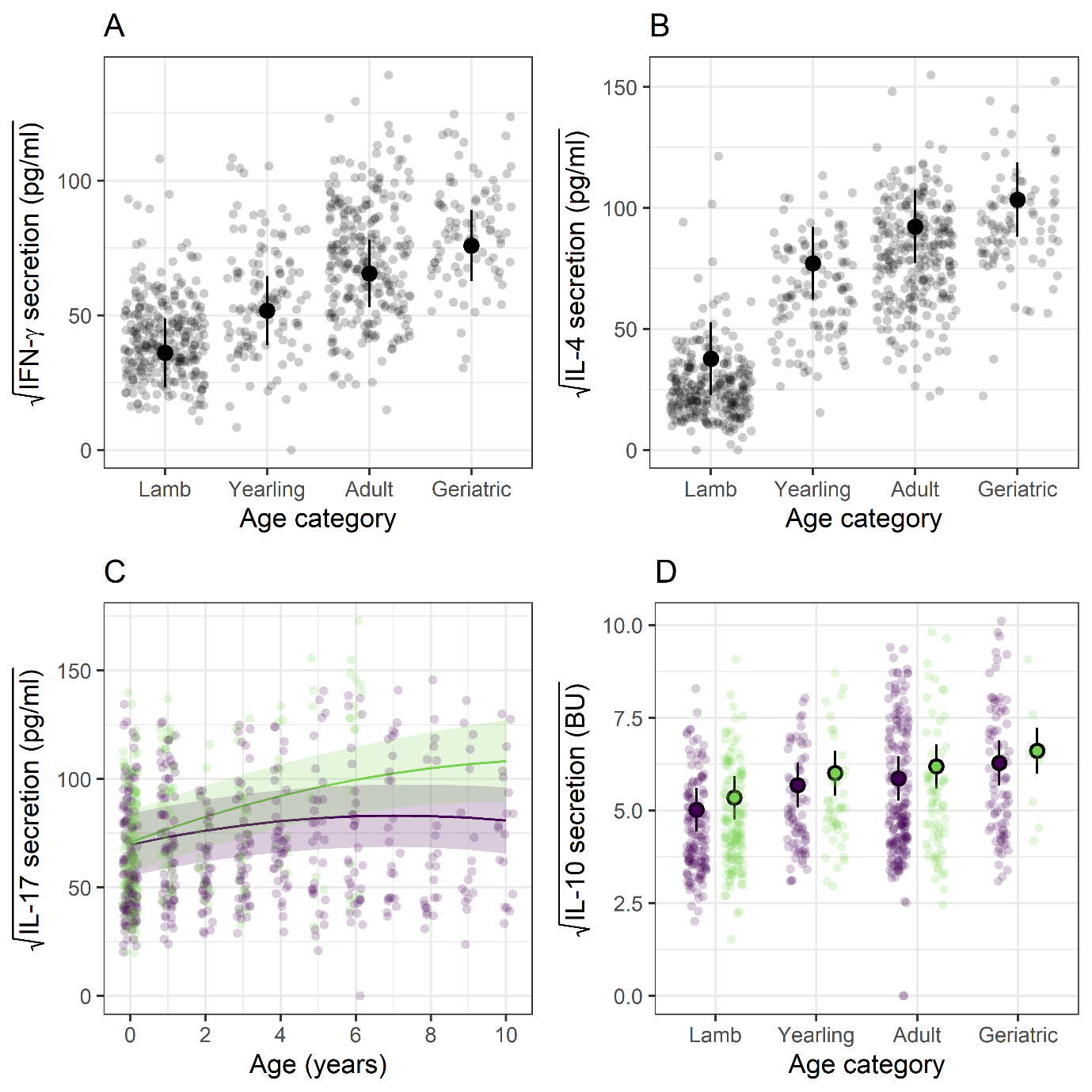


***Figure S4.*** *Age- and/or sex-specific variation in cytokine levels: (A) IFN-γ (Th1); (B) IL-4 (Th2); (C) IL-17A (Th17); and (D) IL-10 (Treg). Points show raw data; large points with bars show estimates ± 95% CI from a model where age/sex were fitted as factors and lines with shaded areas show estimates ± 95% CI from models where age was fitted as a continuous variable. For model details, see Supplementary Table S1. Purple = females and green = males.*


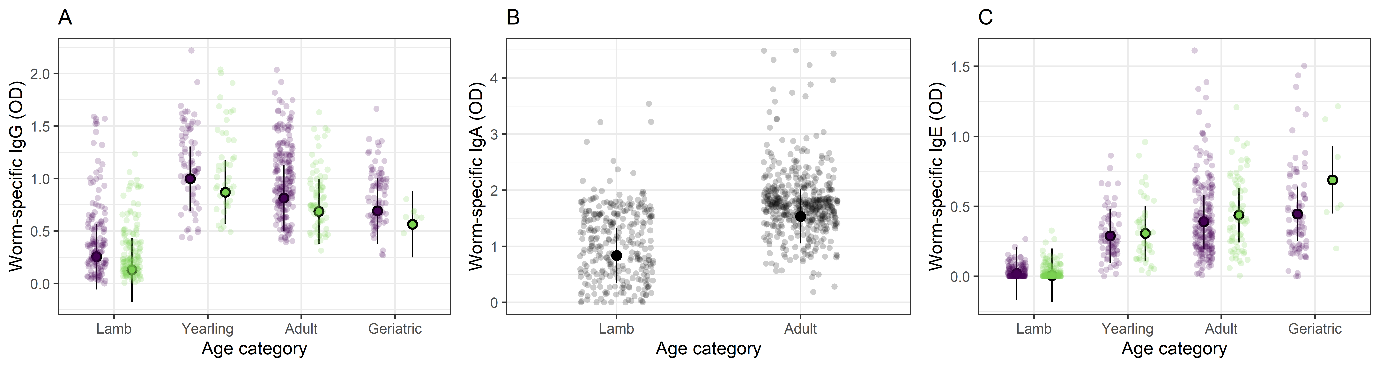


***Figure S5.*** *Age- and sex-specific variation in* T. circumcincta*-specific antibody levels: (A) Tci-IgG; (B) Tci-IgA; (C) Tci-IgE. Points show raw data; large points with bars show estimates ± 95% CI. For model details, see Supplementary Table S1. Red = females and blue = males.*


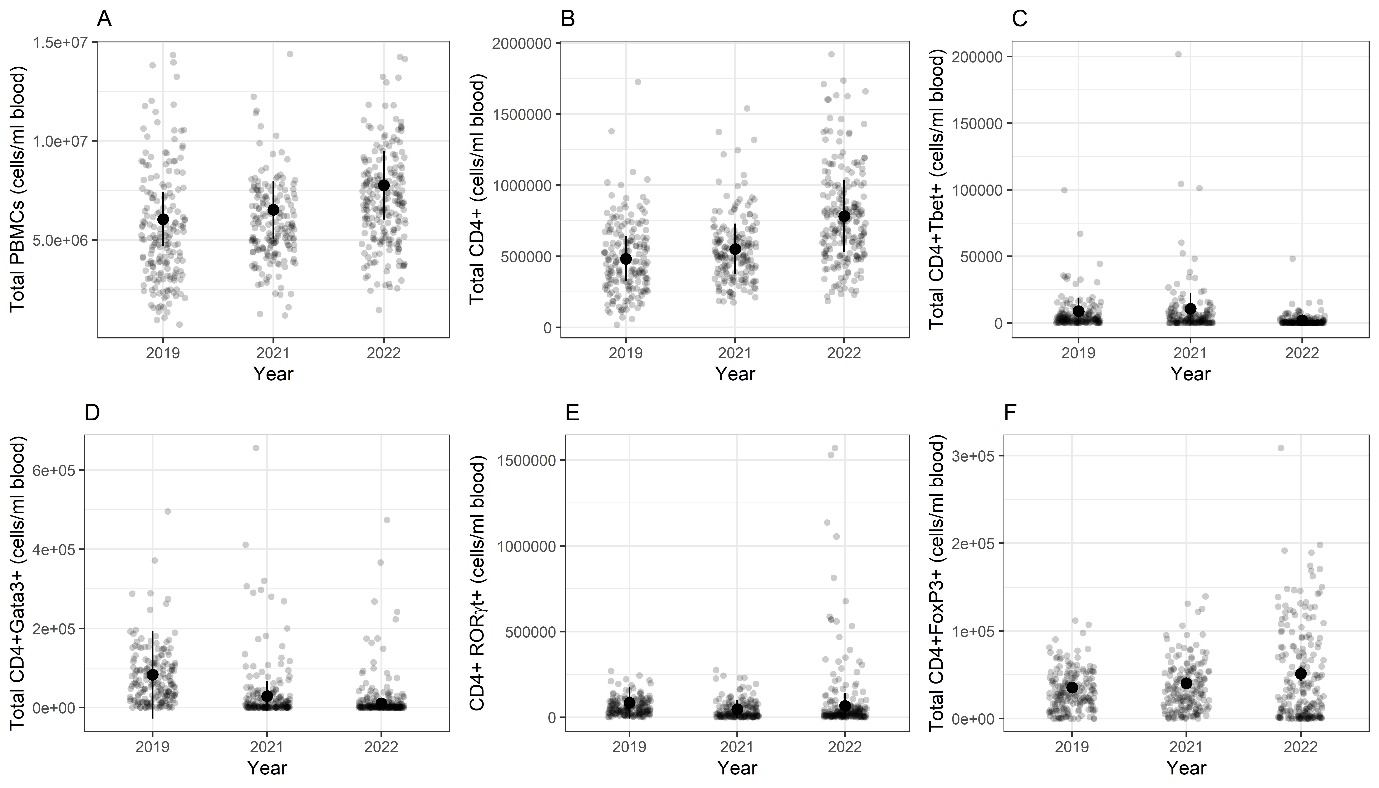


***Figure S6.*** *Year-specific variation in Th cell counts: (A) PBMC; (B) CD4^+^ cells; (C) CD4^+^T-bet^+^ cells (Th1); (D) CD4^+^GATA3^+^ cells (Th2); (E) CD4^+^RORγt^+^ cells (Th17); (F) CD4^+^Foxp3^+^ cells (Treg). Points show raw data; large points show estimates ± 95% CI from the best-fitting model defined in Table S1.*


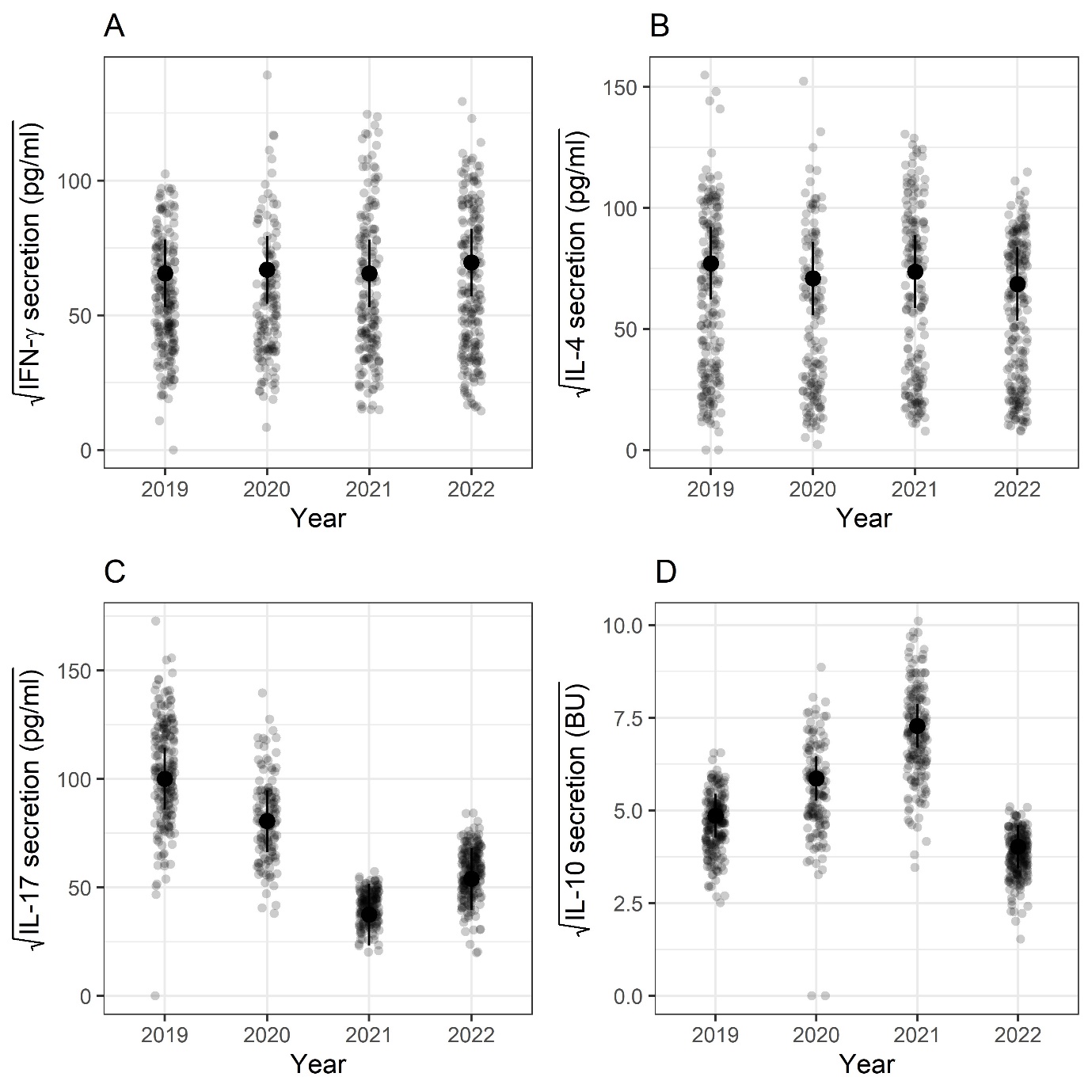


***Figure S7.*** *Year-specific variation in cytokine levels: (A) IFN-γ (Th1); (B) IL-4 (Th2); (C) IL-17A (Th17); and (D) IL-10 (Treg). Points show raw data; large points with bars show estimates ± 95% CI from the best-fitting model in Table S1.*


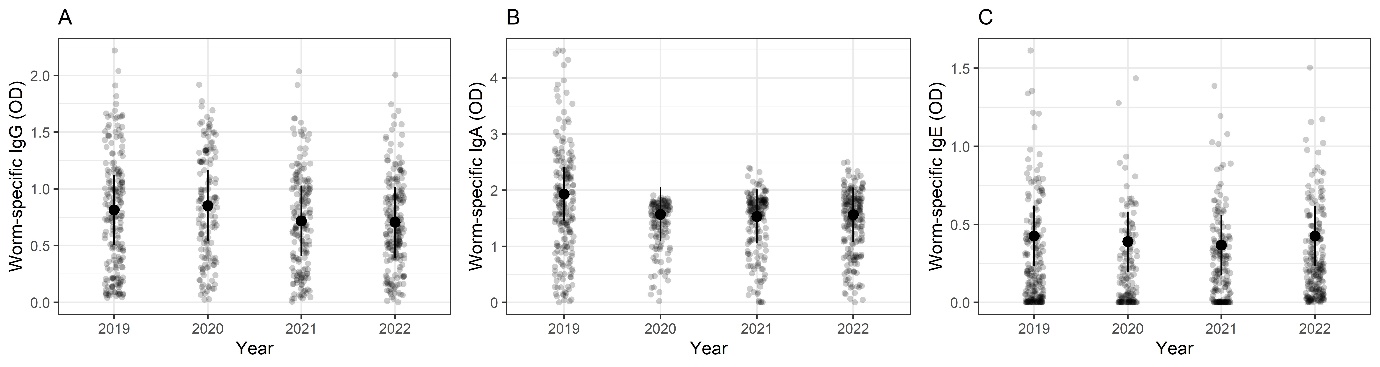


***Figure S8.*** *Year-specific variation in* T. circumcincta*-specific antibody levels: (A) Tci-IgG; (B) Tci-IgA; (C) Tci-IgE. Points show raw data; large points with bars show estimates ± 95% CI. For model details, see Supplementary Table S1.*


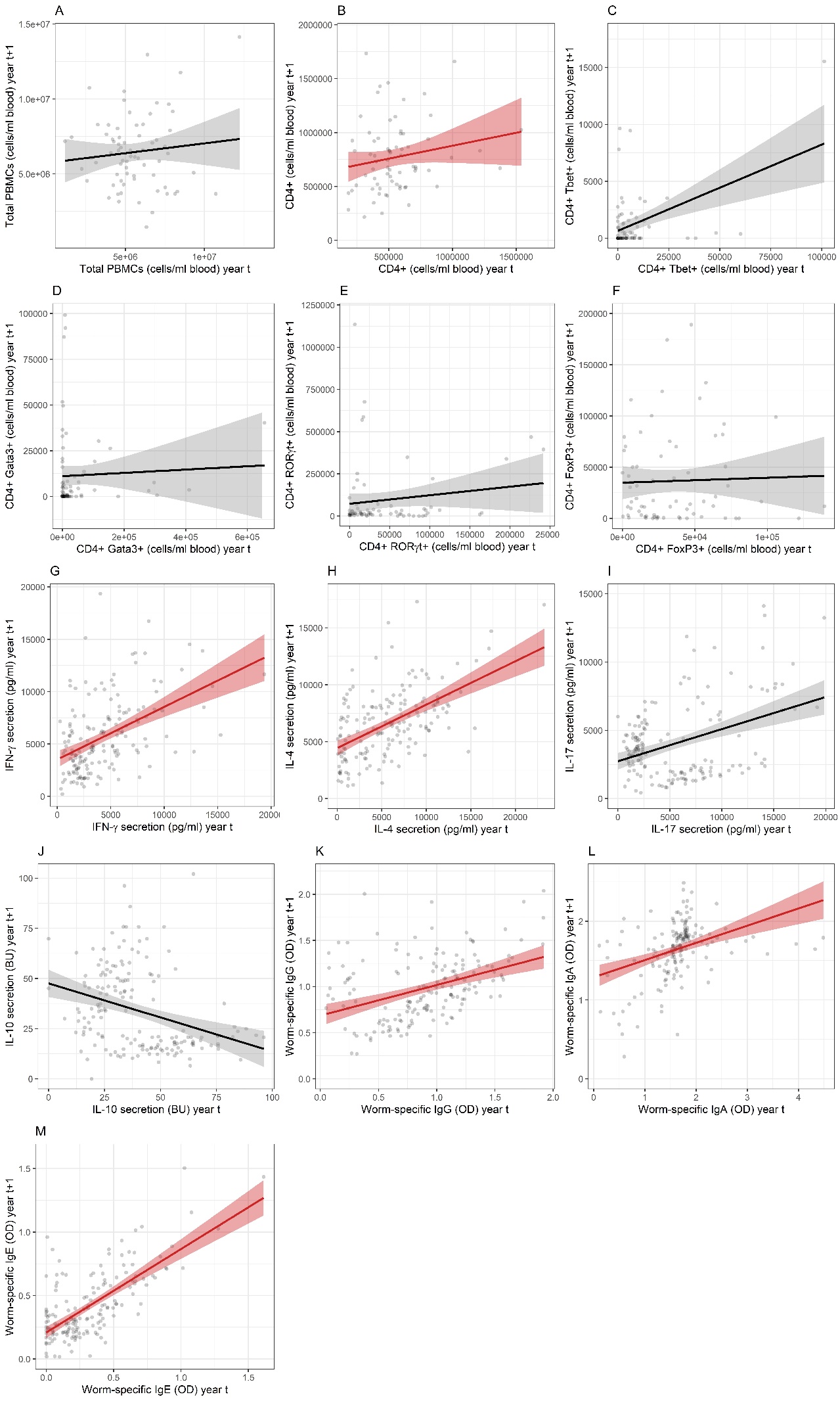


***Figure S9.*** *Correlations between measures of the different immune variables taken in consecutive years. In each plot, the x-axis shows the value of the variable in one year, while the y-axis shows the value of the same variable in the same individual in the subsequent year. Points show raw data and regression line and shaded area show a simple linear regression ±95%CI. Regression lines shaded in red are for those traits that had significant repeatability. Data are presented for illustrative purposes and do not reflect how the repeatability analysis works in practice; however, they illustrate that repeatable traits tend to have stronger positive correlations between successive measures from the same animal. A-F, T-helper cell counts; G-J, cytokines; K-M,* T. circumcincta*-specific antibodies. The different arms of the cell-mediated response represented are Th1 (CD4^+^T-bet^+^, IFN_γ_), Th2 (CD4^+^GATA3^+^, IL-4), Th17 (CD4^+^RORgt^+^, IL-17) and Treg (CD4^+^Foxp3^+^, IL-10).*

| **Model** | **Fixed effects** | **Variance component** | **Trait** | **Estimate** | **L-95%CI** | **U-95%CI** |
| --- | --- | --- | --- | --- | --- | --- |
| 0 | Age(4) + Sex + Year | Individual | σ²(IFN-γ) | 1373791 | 630771 | 1975859 |
|  |  |  | COV(IFN-γ,IL-4) | 597638 | -37838 | 1258081 |
|  |  |  | ρ(IFN-γ,IL-4) | 0.3376 | 0.0379 | 0.6393 |
|  |  |  | σ²(IL-4) | 2045259 | 922193 | 2919349 |
|  |  | Residual | σ²(IFN-γ) | 5106792 | 4305991 | 5834454 |
|  |  |  | COV(IFN-γ,IL-4) | 2914854 | 2221239 | 3610651 |
|  |  |  | ρ(IFN-γ,IL-4) | 0.5381 | 0.4545 | 0.6119 |
|  |  |  | σ²(IL-4) | 5729656 | 4734343 | 6702961 |
| 1 | 0 + FEC | Individual | σ²(IFN-γ) | 1379324 | 666288 | 2044955 |
|  |  |  | COV(IFN-γ,IL-4) | 607284 | -78650 | 1276685 |
|  |  |  | ρ(IFN-γ,IL-4) | 0.3416 | 0.0085 | 0.6262 |
|  |  |  | σ²(IL-4) | 2044297 | 1027109 | 3059001 |
|  |  | Residual | σ²(IFN-γ) | 5101403 | 4307619 | 5943243 |
|  |  |  | COV(IFN-γ,IL-4) | 2911333 | 2124891 | 3668249 |
|  |  |  | ρ(IFN-γ,IL-4) | 0.5382 | 0.4574 | 0.6188 |
|  |  |  | σ²(IL-4) | 5713443 | 4735626 | 6848717 |
| 2 | 0 + weight | Individual | σ²(IFN-γ) | 1390793 | 672050 | 2053371 |
|  |  |  | COV(IFN-γ,IL-4) | 609218 | 14388 | 1373762 |
|  |  |  | ρ(IFN-γ,IL-4) | 0.3427 | 0.0233 | 0.6126 |
|  |  |  | σ²(IL-4) | 2017111 | 964607 | 2938380 |
|  |  | Residual | σ²(IFN-γ) | 5072831 | 4326336 | 5959002 |
|  |  |  | COV(IFN-γ,IL-4) | 2865807 | 2113195 | 3548727 |
|  |  |  | ρ(IFN-γ,IL-4) | 0.5323 | 0.4552 | 0.6157 |
|  |  |  | σ²(IL-4) | 5693205 | 4792689 | 6758309 |
| 3 | 0 + FEC + weight | Individual | σ²(IFN-γ) | 1400898 | 752001 | 2044503 |
|  |  |  | COV(IFN-γ,IL-4) | 618259 | 10465 | 1343363 |
|  |  |  | ρ(IFN-γ,IL-4) | 0.3480 | 0.0498 | 0.6400 |
|  |  |  | σ²(IL-4) | 2025321 | 938649 | 2990815 |
|  |  | Residual | σ²(IFN-γ) | 5079802 | 4287397 | 5843960 |
|  |  |  | COV(IFN-γ,IL-4) | 2875001 | 2115198 | 3566836 |
|  |  |  | ρ(IFN-γ,IL-4) | 0.5333 | 0.4479 | 0.6145 |
|  |  |  | σ²(IL-4) | 5699795 | 4672493 | 6695279 |

***Table S2.*** *Variance components estimates from bivariate linear mixed-effects models of IFN-γ and IL-4. The four models differ only in their fixed effects structure. Variance components are as follows: σ²(IFN-γ) = variance associated with IFN-γ; COV(IFN-γ,IL-4) = covariance between IFN-γ (Th1) and IL-4 (Th2); ρ(IFN-γ,IL-4) = correlation between IFN-γ and IL-4; σ²(IL-4) = variance associated with IL-4. L-95%CI and U-95%CI = lower and upper 95% confidence intervals.*

| **Variable** | **Model** | **Estimate** | **SE** | **χ²** | **P** |
| --- | --- | --- | --- | --- | --- |
| **PBMC** | Main effect | -0.0154 | 0.0515 | 0.09 | 0.765 |
|  | Age interaction | 0.0942 | 0.0991 | 0.90 | 0.343 |
|  | Year interaction | NA | NA | 2.48 | 0.289 |
| **CD4+** | Main effect | -0.0209 | 0.0526 | 0.16 | 0.692 |
|  | Age interaction | 0.0125 | 0.0922 | 0.02 | 0.892 |
|  | Year interaction | NA | NA | 4.25 | 0.120 |
| **CD4+ T-bet+**  **(Th1)** | Main effect | -0.0233 | 0.0454 | 0.25 | 0.617 |
|  | **Age interaction** | **0.2316** | **0.1222** | **3.94** | **0.047** |
|  | Year interaction | NA | NA | 3.42 | 0.181 |
| **CD4+ GATA3+**  **(Th2)** | Main effect | -0.0450 | 0.0481 | 0.85 | 0.357 |
|  | Age interaction | 0.1722 | 0.0906 | 3.52 | 0.061 |
|  | Year interaction | NA | NA | 5.63 | 0.060 |
| **CD4+ RORγt+**  **(Th17)** | Main effect | 0.0377 | 0.0488 | 0.64 | 0.423 |
|  | Age interaction | -0.1270 | 0.1096 | 1.31 | 0.252 |
|  | Year interaction | NA | NA | 0.52 | 0.770 |
| **CD4+ Foxp3+**  **(Treg)** | Main effect | -0.0203 | 0.0445 | 0.21 | 0.650 |
|  | Age interaction | -0.0140 | 0.0887 | 0.02 | 0.875 |
|  | Year interaction | NA | NA | 1.66 | 0.437 |
| **IFN-γ**  **(Th1)** | Main effect | -0.0665 | 0.0483 | 1.87 | 0.171 |
|  | Age interaction | 0.0181 | 0.1518 | 0.01 | 0.905 |
|  | Year interaction | NA | NA | 6.60 | 0.086 |
| **IL-4**  **(Th2)** | **Main effect** | **-0.1881** | **0.0590** | **9.89** | **0.002** |
|  | Age interaction | 0.1584 | 0.2352 | 0.43 | 0.511 |
|  | Year interaction | NA | NA | 7.41 | 0.060 |
| **IL-17A**  **(Th17)** | Main effect | 0.0477 | 0.0714 | 0.45 | 0.503 |
|  | Age interaction | -0.0486 | 0.1031 | 0.22 | 0.636 |
|  | Year interaction | NA | NA | 2.03 | 0.566 |
| **IL-10**  **(Treg)** | Main effect | 0.0055 | 0.0723 | 0.01 | 0.939 |
|  | Age interaction | 0.1191 | 0.1085 | 1.19 | 0.275 |
|  | Year interaction | NA | NA | 0.48 | 0.923 |
| **IgG** | Main effect | -0.0290 | 0.0567 | 0.26 | 0.610 |
|  | Age interaction | 0.6615 | 0.0942 | 2.52 | 0.112 |
|  | Year interaction | NA | NA | 0.51 | 0.916 |
| **IgA** | Main effect | -0.0027 | 0.0500 | 0.00 | 0.957 |
|  | Age interaction | 0.6036 | 0.0774 | 0.27 | 0.601 |
|  | Year interaction | NA | NA | 1.70 | 0.637 |
| **IgE** | **Main effect** | **-0.1159** | **0.0515** | **4.89** | **0.027** |
|  | Age interaction | 0.6357 | 0.3694 | 0.01 | 0.912 |
|  | **Year interaction** | **NA** | **NA** | **8.71** | **0.033** |

***Table S3.*** *Results of GLMMs of associations between strongyle FEC and each immunological parameters. Estimates and test statistics are shown for models where only the variable indicated was included in the model; interaction estimates show difference in slope between ages (lambs and adults); year interaction estimates for year not shown to limit space use. Associations significant at α = 0.05 are highlighted in bold.*

| **Variable** | **Model** | **Estimate** | **SE** | **χ²** | **P** |
| --- | --- | --- | --- | --- | --- |
| **PBMCs** | Main effect | 0.0354 | 0.0546 | 0.42 | 0.516 |
|  | Age interaction | 0.0346 | 0.1062 | 0.11 | 0.745 |
|  | Year interaction | NA | NA | 0.23 | 0.893 |
| **CD4+** | Main effect | 0.0204 | 0.0572 | 0.13 | 0.722 |
|  | Age interaction | -0.0438 | 0.1038 | 0.18 | 0.673 |
|  | Year interaction | NA | NA | 2.21 | 0.331 |
| **CD4+ T-bet+**  **(Th1)** | Main effect | -0.0085 | 0.0492 | 0.03 | 0.864 |
|  | Age interaction | 0.1304 | 0.1286 | 1.05 | 0.305 |
|  | Year interaction | NA | NA | 1.67 | 0.433 |
| **CD4+ GATA3+**  **(Th2)** | Main effect | 0.0033 | 0.0552 | 0.00 | 0.953 |
|  | Age interaction | 0.1891 | 0.1022 | 3.35 | 0.067 |
|  | Year interaction | NA | NA | 3.79 | 0.151 |
| **CD4+ RORγt+**  **(Th17)** | Main effect | -0.0082 | 0.0513 | 0.03 | 0.874 |
|  | Age interaction | 0.0134 | 0.1039 | 0.02 | 0.897 |
|  | Year interaction | NA | NA | 0.32 | 0.854 |
| **CD4+ Foxp3+**  **(Treg)** | Main effect | 0.0130 | 0.0512 | 0.06 | 0.800 |
|  | Age interaction | -0.0807 | 0.1024 | 0.62 | 0.430 |
|  | Year interaction | NA | NA | 1.66 | 0.436 |
| **IFN-γ**  **(Th1)** | Main effect | **-0.1146** | **0.0536** | **4.51** | **0.034** |
|  | Age interaction | 0.2818 | 0.1743 | 2.54 | 0.111 |
|  | **Year interaction** | **NA** | **NA** | **7.92** | **0.048** |
| **IL-4**  **(Th2)** | Main effect | -0.0582 | 0.0643 | 0.81 | 0.367 |
|  | Age interaction | -0.1322 | 0.2481 | 0.29 | 0.588 |
|  | **Year interaction** | **NA** | **NA** | **10.73** | **0.013** |
| **IL-17A**  **(Th17)** | Main effect | -0.0171 | 0.0817 | 0.04 | 0.834 |
|  | Age interaction | -0.0010 | 0.1149 | 0.00 | 0.993 |
|  | Year interaction | NA | NA | 0.19 | 0.979 |
| **IL-10**  **(Treg)** | Main effect | -0.0825 | 0.0808 | 1.04 | 0.308 |
|  | Age interaction | 0.1183 | 0.1167 | 1.02 | 0.313 |
|  | Year interaction | NA | NA | 1.60 | 0.658 |
| **IgG** | Main effect | -0.0290 | 0.0654 | 0.20 | 0.658 |
|  | Age interaction | 0.1420 | 0.1292 | 1.20 | 0.274 |
|  | Year interaction | NA | NA | 2.72 | 0.437 |
| **IgA** | Main effect | -0.0623 | 0.0551 | 1.27 | 0.260 |
|  | Age interaction | -0.0098 | 0.1090 | 0.01 | 0.929 |
|  | Year interaction | NA | NA | 1.78 | 0.619 |
| **IgE** | **Main effect** | **-0.1420** | **0.0650** | **4.68** | **0.031** |
|  | Age interaction | 0.1270 | 0.4259 | 0.09 | 0.766 |
|  | **Year interaction** | **NA** | **NA** | **8.65** | **0.034** |

***Table S4.*** *Results of GLMMs of associations between coccidian FOC and each immunological parameters. Estimates and test statistics are shown for models where only the variable indicated was included in the model; interaction estimates show difference in slope between ages (lambs and adults); year interaction estimates for year and not shown to limit space use. Associations significant at α = 0.05 are highlighted in bold.*

| **Variable** | **Records** | **Model** | **Estimate** | **SE** | **χ²** | **P** |
| --- | --- | --- | --- | --- | --- | --- |
| **Weight** | 267 | **Main** | **0.2473** | **0.0592** | **19.84** | **<0.001** |
|  |  | Interaction | NA | NA | 4.17 | 0.243 |
| **FEC** | 242 | **Main** | **-0.0017** | **0.0007** | **6.49** | **0.011** |
|  |  | Interaction | NA | NA | 2.61 | 0.455 |
| **FOC** |  | Main | 0.0000 | 0.0000 | 0.03 | 0.865 |
|  |  | **Interaction** | **NA** | **NA** | **15.88** | **0.001** |
| **PBMC** | 199 | Main | 0.0000 | 0.0000 | 1.28 | 0.258 |
|  |  | Interaction | NA | NA | 2.22 | 0.330 |
| **CD4+** |  | Main | 0.0000 | 0.0000 | 1.33 | 0.249 |
|  |  | Interaction | NA | NA | 2.74 | 0.254 |
| **CD4+ T-bet+**  **(Th1)** |  | Main | 0.0000 | 0.0000 | 0.02 | 0.885 |
|  |  | Interaction | NA | NA | 0.70 | 0.706 |
| **CD4+ GATA3+**  **(Th2)** |  | Main | 0.0000 | 0.0000 | 0.17 | 0.682 |
|  |  | **Interaction** | **NA** | **NA** | **6.03** | **0.049** |
| **CD4+ RORγt+**  **(Th17)** |  | Main | 0.0000 | 0.0000 | 0.37 | 0.542 |
|  |  | Interaction | NA | NA | 4.76 | 0.092 |
| **CD4+ Foxp3+**  **(Treg)** |  | Main | 0.0000 | 0.0000 | 1.17 | 0.279 |
|  |  | Interaction | NA | NA | 2.90 | 0.234 |
| **IFN-γ**  **(Th1)** | 273 | Main | 0.0000 | 0.0000 | 0.15 | 0.702 |
|  |  | Interaction | NA | NA | 1.39 | 0.708 |
| **IL-4**  **(Th2)** |  | Main | 0.0000 | 0.0000 | 0.30 | 0.583 |
|  |  | Interaction | NA | NA | 5.94 | 0.114 |
| **IL-17A**  **(Th17)** |  | Main | 0.0000 | 0.0000 | 1.08 | 0.298 |
|  |  | Interaction | NA | NA | 4.33 | 0.228 |
| **IL-10**  **(Treg)** |  | Main | -0.1290 | 0.0167 | 0.60 | 0.438 |
|  |  | Interaction | NA | NA | 5.74 | 0.125 |
| **IgG** | 273 | Main | 0.6557 | 0.4130 | 2.59 | 0.108 |
|  |  | Interaction | NA | NA | 5.77 | 0.124 |
| **IgA** | 273 | Main | -0.0818 | 0.2005 | 0.17 | 0.683 |
|  |  | Interaction | NA | NA | 1.80 | 0.616 |
| **IgE** | 273 | **Main** | **5.8400** | **2.8100** | **4.70** | **0.030** |
|  |  | Interaction | NA | NA | 2.11 | 0.550 |

***Table S5.*** *Results from binomial generalized linear models of lamb survival. “Records” shows the number of lambs included in the analysis for each variable while estimates, SE, Χ² and P refer to the term being tested, which is either the main effect or the interaction with year “Interaction”). Terms were initially fitted individually and then terms highlighted in bold were deemed to be significant and moved together to a final model where their independent effects were assessed.*
